# High‐Sensitive Mass Cytometry Metal Tags Based on Noble Metal Nanoparticles Assisting Acute Myeloid Leukemia Immune Checkpoint Profiling

**DOI:** 10.1002/advs.74529

**Published:** 2026-03-02

**Authors:** Zhizhou Liu, Yu Yang, Lingfei Qiu, Yi Yang, Jingrui Li, Juan Yue, Yinghui Li, Yingdai Gao, Pengli Bai

**Affiliations:** ^1^ Suzhou Institute of Biomedical Engineering and Technology Chinese Academy of Sciences Suzhou Jiangsu China; ^2^ Tianjin Guoke Medical Engineering and Technology Development Co., Ltd Tianjin China; ^3^ School of Precision Instrument and Opto‐Electronics Engineering Tianjin University Tianjin China; ^4^ Department of Chemistry College of Sciences Shanghai University Shanghai China; ^5^ State Key Laboratory of Experimental Hematology National Clinical Research Center for Blood Diseases, Haihe Laboratory of Cell Ecosystem, Institute of Hematology & Blood Diseases Hospital Chinese Academy of Medical Sciences and Peking Union Medical College Tianjin China; ^6^ Tianjin Institutes of Health Science Tianjin 301600 China

**Keywords:** AML immune checkpoint profiling, mass cytometry, noble metal nanoparticles, single cell analysis

## Abstract

Mass cytometry is optimal for acute leukemia immune checkpoint profiling. However, current commercial metal‐chelating polymer (MCP) metal tags limit its sensitivity due to low metal loading capacity. We report a novel synthetic strategy employing antibody‐conjugated gold or platinum nanoparticles (AuNP/PtNP) for high‐dimensional single‐cell immunophenotyping in both single cell suspension mass cytometry and imaging mass cytometry. Extremely low‐dose AuNP (34 NPs/cell) exhibited MCP‐comparable sensitivity, while high doses enabled up to 48‐fold signal amplification. Furthermore, integration of the detection of low‐abundance TIGIT and OX40 by AuNP/PtNP tags and other 22 biomarkers by MCP tags, three significantly distinct T cell subpopulations in healthy donor and acute myeloid leukemia samples were identified, providing new targets and intervention strategies for the diagnosis and treatment of acute myeloid leukemia. The proposed strategies demonstrate that noble metal NP‐based tags show promising potential in detection of low‐abundance cell surface biomarkers and enhance MC implementation in both biomedical and clinical applications.

## 1 Introduction

Immune checkpoints represent critical regulators of antitumor immune responses, and their blockade on immune effector cells can reinvigorate anti‐tumor immunity [1, 2, 3]. Emerging evidence demonstrates that combined therapy using programmed cell death protein 1 (PD‐1) and cytotoxic T‐lymphocyte antigen 4 (CTLA‐4) inhibitors with hypomethylating agents (HMAs) may benefit patients with relapsed acute myeloid leukemia (AML) and high‐risk myelodysplastic syndrome (MDS) [4, 5]. However, a substantial proportion of AML cases remain refractory to these targeted interventions, underscoring the urgent need for comprehensive understanding of immune checkpoints and identification of novel therapeutic targets or combinatorial blockade strategies to enhance the efficacy of immune checkpoint‐based therapies for AML. Mass cytometry (MC) represents a cutting‐edge bioanalytical platform enabling high‐dimensional profiling at single‐cell resolution [6, 7, 8, 9, 10, 11], and is optimal for acute leukemia immune checkpoint profiling. By utilizing heavy isotope‐labeled antibodies (Abs) to identify different subpopulations of cells, it overcomes the limitations of conventional fluorescence flow cytometry resolution caused by spectral overlap, enabling the simultaneous detection of about 50 biomarkers from individual cells from a single sample [12, 13, 14, 15, 16]. However, the technological constraints of current MC metal‐tagging systems have hampered their broader application in both biomedical research and clinical practice.

The current MC metal tags are typically metal‐chelating polymers (MCPs), which carry only 20–50 metal atoms per tag [17, 18, 19, 20]. Given that MC's ion transmission rates range from 10^−5^ to 10^−4^, hundreds of MCPs must bind to a single cell for detectable signal generation [21, 22]. Several nanoparticle (NP) materials have been explored for loading more heavy metal atoms to increase MC sensitivity so far [23, 24, 25, 26, 27, 28]. Disappointingly, although these NPs contain hundreds or thousands of times atoms more than MCP reagents, only limited amplifications for the MC detection signal are achieved. For example, 40 nm Ag NP containing 2 × 10^6^ Ag atoms, which is 10^4^ fold higher than MCP reagents, represents similar sensitivity with MCPs [29]. The most successful NP‐based metal tag is polyethylene glycol (PEG) modified 12 nm‐NaHoF_4_, which afforded 30‐fold signal enhancement in the direct detection of CD14 molecules on THP‐1 cells [30]. Moreover, the utilization of separated high‐purity metal isotopes for material synthesis is highly uneconomical as hundreds of milligrams or even grams scaled starting materials are required in the synthesis of these NPs [13, 30, 31]. Besides, the low yields of these NPs further reduce isotopic utilization. Exploring novel metal tags synthetic strategy with high isotopic utilization rate is still challenge.

Nanoscale aggregates of noble metals enable a much higher atom content per particle, rendering them excellent metal tag candidates. 197Au is quite suitable for MC single cell analysis due to its 100% natural abundance and low biological occurrence. In previous research gold nanoparticle (AuNP) can be endocytosed by cells and detected by MC [32]. Streptavidin‐conjugated AuNP can serve as reporter for the cytokines bead‐based immunoassays [33, 34, 35]. The application of Abs‐conjugated AuNP in single cell biomarkers detection has not yet been explored, which is likely attributable to the complex microenvironment at the cell surface. Winnik reported a dipicolylamine‐based polymer to chelate Pt ion and introduce Pt elements into MC [19, 36, 37]. However, the large polymer backbone limited the Pt capacity, resulting in similar detection sensitivity with MCP reagents. Platinum nanoparticle (PtNP) not only contains large amount of Pt atoms, but also possesses anti‐fouling interface performance, representing an excellent candidate for MC single‐cell immunophenotyping reagent. More importantly, the synthesis of AuNP (or PtNP) is via a well‐established reduction of HAuCl_4_ (or H_2_PtCl_6_). The yield is almost 100% [38, 39], and the synthetic scale can be precisely regulated, maximizing the utilization rate of isotopic materials.

**SCHEME 1:**
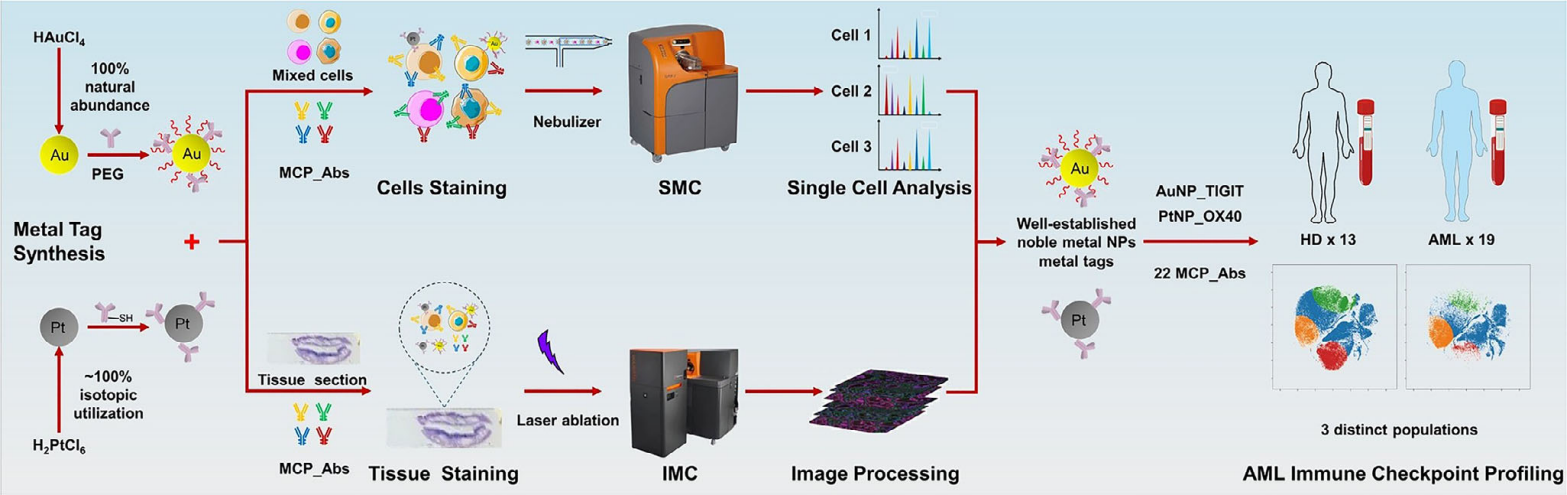
Schematic illustration of synthesizing noble metal NPs based mass tags and applying AuNP/PtNP/MCP conjugated Abs cocktail for SMC and IMC assays.

Herein, we present a new strategy to prepare MC single cell metal tags based on gold and platinum nanoparticles via simple reduction synthesis with ∼100% isotopic utilization, showing much higher sensitivity than the commercial MCP metal tags and excellent compatibility with other MC reagents in both single cell suspension mass cytometry (SMC) and imaging mass cytometry (IMC). The overall experiment flow is schematically illustrated in Scheme 1. These noble metal NPs were coupled with antibodies and further modified with PEG derivatives of varying lengths and different functional groups to improve their specific binding performance with cells. The Au/Pt NP‐Abs conjugates were subsequently employed for phenotypic subtyping of T‐cell subpopulations based on high versus low expression levels to test their sensitivity and specificity. Finally, 19 AML and 13 healthy donor (HD) samples were analyzed using AuNP and PtNP labeled anti‐TIGIT and OX40 antibodies in combination with 22 MCP tags, identifying three significantly distinct T cell subpopulations. This work demonstrates that noble metal NPs based tags possess outstanding potential for MC implementation in practical biomedical applications.

## 2 Results and Discussion

### 2.1 Preparation and Characterization of 197AuNP_Abs

**FIGURE 1:**
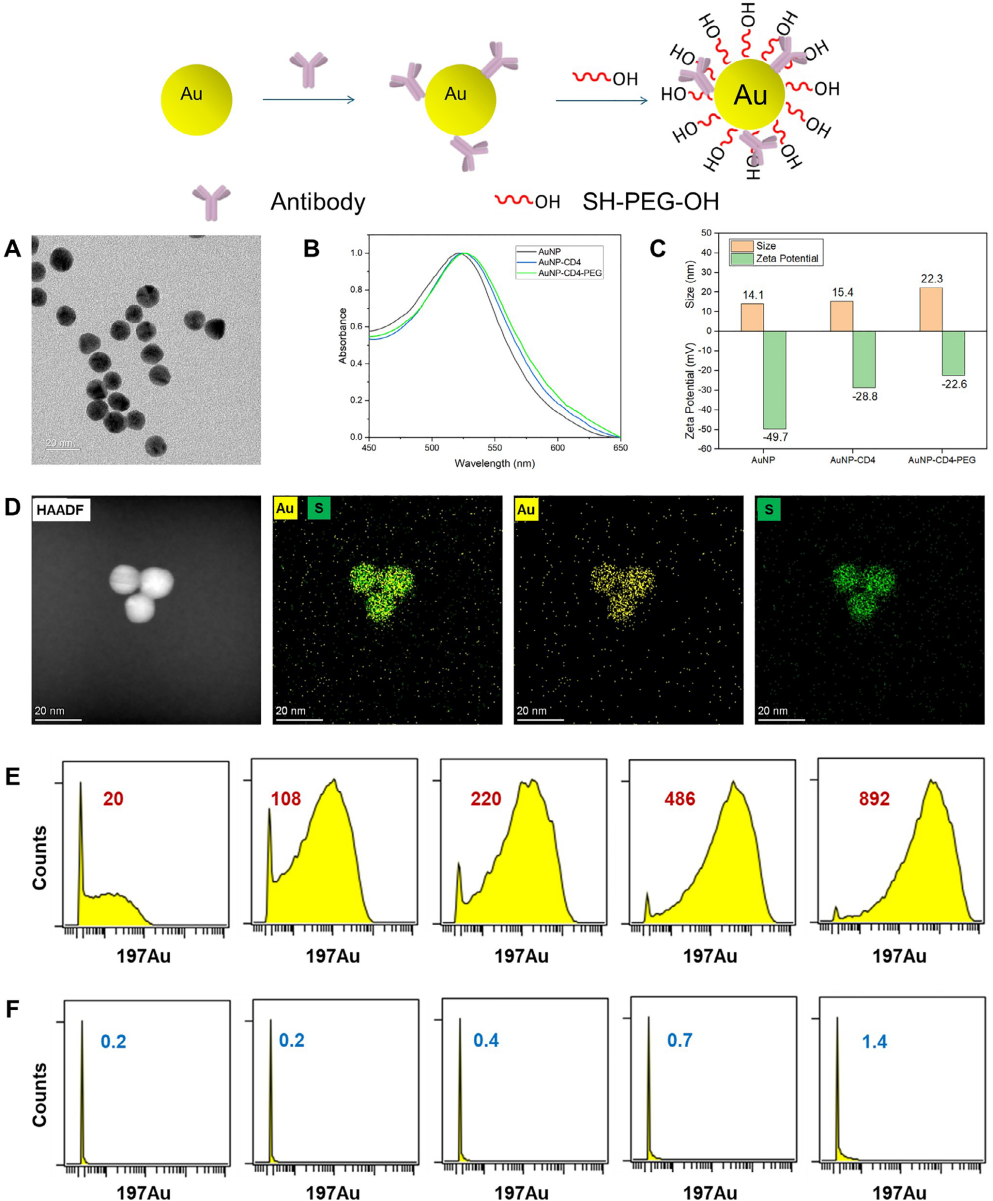
The synthesis and characterization of AuNP‐based metal tags. (A) TEM image of AuNP; (B) UV–vis spectrum of AuNP, 197AuNP_CD4 and 197AuNP_CD4‐PEG; (C) DLS size and Zeta potential of AuNP, 197AuNP_CD4 and 197AuNP_CD4‐PEG; (D) High‐resolution EDX mapping images for the distribution of Au and S in the 197AuNP_CD4, respectively; Histograms of 197Au counts on PBMCs (E) AuNP and (F) AuNP‐PEG (3.4 K, 17 K, 34 K, 68 K, 136 K NPs/cell, respectively). Depending on the transmission coefficient of Au for mass cytometer (on the order of 10^−5^), a single signal count corresponds to approximately 10^5^ Au atoms conjugated per cell.

The AuNP was synthesized via the simple reduction of following a reported literature [40]. As shown in the transmission electron microscopy (TEM) image (Figure 1A), the AuNPs were monodispersed spheres with size of about 10 nm. The AuNP‐based MC marker was prepared through the grafting of the antibodies onto the AuNP surface via electrostatic adsorption and hydrophobic interaction, following with functionalization with PEG. These processes were monitored by UV‐vis spectroscopy (UV–vis) and Dynamic Light Scattering (DLS) detection as shown in Figure 1B,C. AuNP represent its characteristic absorption peak at 528 nm, which was shifted to 526 and 522 nm after the conjugation with anti‐CD4 antibody and PEG modification, respectively. The corresponding hydration radius increased from 14.1 to 22.3 nm while the zeta potential increased from −49.7 to −22.6 mV. The conjugation of AuNP was also confirmed by the high resolution energy dispersive X‐ray (EDX) spectroscopy in Figure 1D, where S element was derived from Au─S bond of AuNP‐antibody conjugation [41]. The number of CD4 antibody per AuNP was estimated via an indirect approach. The amount of unbound antibody in the supernatant after the conjugation reaction was determined by a Bradford protein assay after particle sedimentation. The average number of antibodies per AuNP was calculated to be about 1.4 (The calculation was described in experiment section). In order to evaluate the non‐specific binding (NSB) performance between AuNP and cells, different dose of AuNP (measured by NanoSight, Figure S1) were used to stain peripheral blood mononuclear cells (PBMCs). As shown in Figure 1E, trace amount of AuNP resulted high MC signals, while the modification of thiol and hydroxyl functionalized PEG significantly reduced the NSB level. Such performances suggest that AuNP modified by PEG was a promising candidate of MC metal tag.

To determine the specificity of AuNP metal tag, the binding behavior in PBMCs was tested by staining with 89Y‐MCP_CD45, 156Gd‐MCP_CD3, 197AuNP_CD4, and 146Nd‐MCP_CD8. 159Tb‐MCP_CD4 was used as control and the gating strategy was shown in Figure S2. To our surprise, extremely low dose of unmodified 197AuNP_CD4 (34 NPs/cell) can clearly distinguish the CD4^+^/^−^ T cells populations (Figure S3A). To be noted, in order to achieve similar sensitivity, other NPs based metal tags required much higher dose (130, 1500, 15 k NPs/cell for NaHoF_4_, Zr‐MOF, MPF, respectively) [13, 30, 31]. We estimate that each AuNP with a diameter of 10 nm contains 31 000 Au atoms, which is same level with reported 12 nm NaHoF_4_ but much less than 40 nm Zr‐MOF and AgNP. The most possible explain is that the antibody was conjugated with AuNP via simple adsorption and hydrophobic interaction, which is more efficient than covalent coupling as it can avoid lots of side reactions occurred during conjugation. when lower dose of 197AuNP_CD4 (3.4 NPs/cell) was used to stain PBMCs, only half of the CD4 positive T cells were successfully identified. When the dose of 197AuNP_CD4 used was increased to 3.4 K, the CD4^+^/^−^ T cells populations can be distinguished, while the specific binding (SB, 197AuNP_CD4 specific binding to CD4^+^ T cells) intensity was 665, while the NSB (197AuNP_CD4 non‐specific binding to CD4^−^ T cells) intensity was 3.4. Accordingly, the SB intensity of 159Tb‐MCP_CD4 was 303, while the NSB intensity was 1.1 (Figure S3B). However, when higher doses of 197AuNP_CD4 were used to stain PBMCs, the NSB level of AuNP raised subsequently. From the dot plots, heatmap, and histograms in Figures S3AC and S4, we can see that when the dose was increased to 17 K NPs/cell or higher, the SB signal became much higher than MCP. However, the corresponding NSB signal also increased and even form an elongated scatter profile in the CD4^−^ T cell population. Although higher dose of 197AuNP_CD4 can also distinguish CD4^+^/CD8^+^ T cell populations, the pattens of CD8^+^ T cell population become elongated, indicating a high NSB level (Figure S5). The mean intensity of SB and NSB was shown in Figure S3D, and the corresponding SB/NSB ratio was shown in Figure S3E. The dose of 17 K NPs/cell represent a SB/NSB ratio of 236, which is lower than that of MCP (276) and NaHoF_4_ (570) [30] based metal tags, but higher than that of MPF (67) [13], Zr‐MOF (75) [31], and TaO_2_ (200) [28] based metal tags, indicating high specificity of AuNP based metal tags.

**FIGURE 2:**
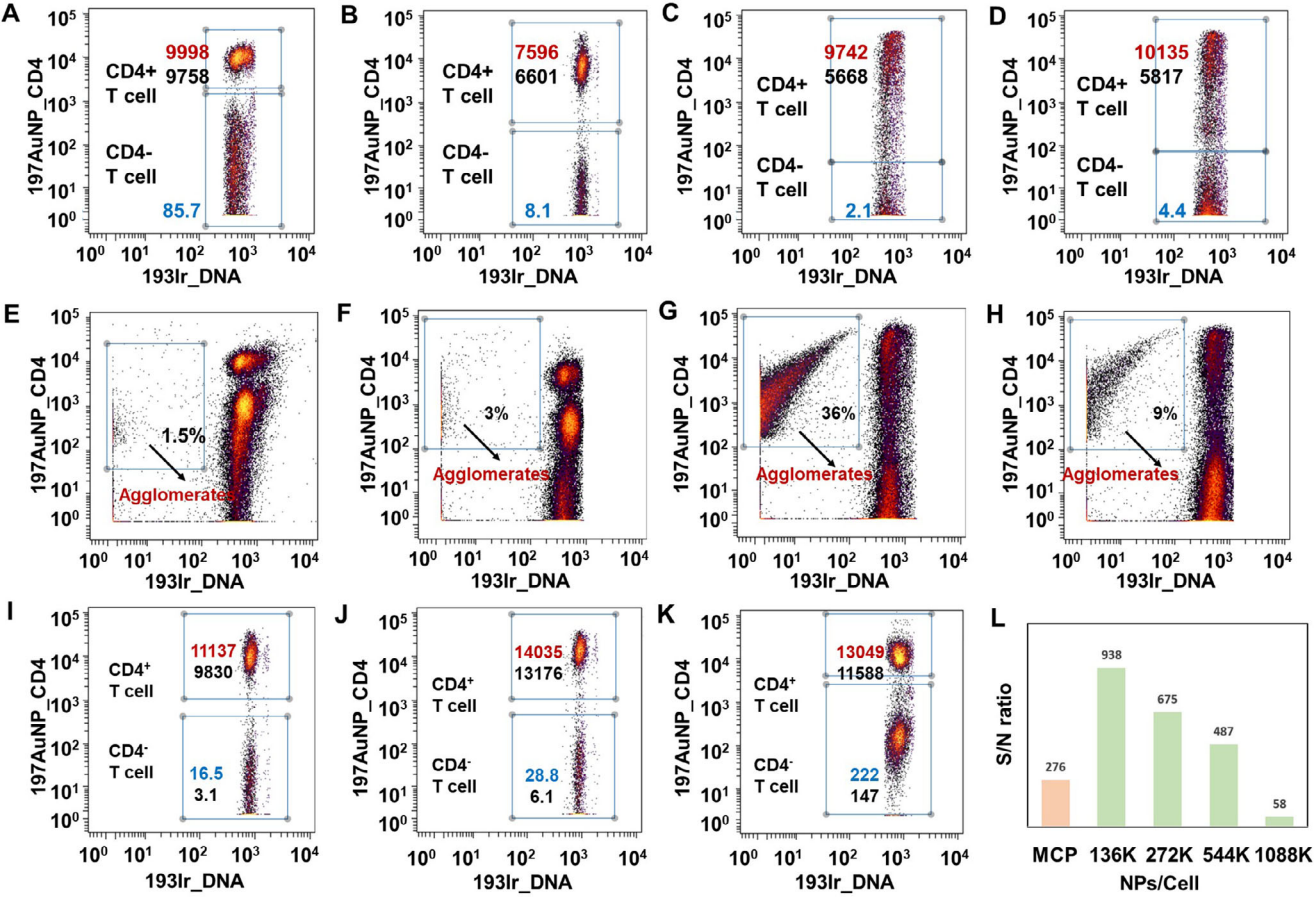
The dot plots of T cells stained by (A) 197AuNP_CD4; (B) 197AuNP_CD4‐PEG_2000_‐OH; (C) 197AuNP_CD4‐PEG_2000_‐NH_2_ and (D) 197AuNP_CD4‐PEG_2000_‐COOH at 136 K NPs/cell concentration; (E‐H) dot plots of ungated population stained by197AuNP_CD4, 197AuNP_CD4‐PEG_2000_‐OH, 197AuNP_CD4‐PEG_2000_‐NH_2_ and 197AuNP_CD4‐PEG_2000_‐COOH; The dot plots of T cells stained by 197AuNP_CD4‐PEG_2000_‐OH at (I) 272 K, (J) 544 K and (K) 1088K NPs/cell concentration; (L) S/N ratios of T cells stained by different concentrations of 197AuNP_CD4‐PEG_2000_‐OH.

In order to maximize the detection sensitivity, thiol‐functionalized PEG with different groups were used to modify 197AuNP_CD4 to lower the NSB between NPs and cells. As shown in Figure S6, the SH‐PEG_2000_‐OH can be successfully modified to 197AuNP_CD4 while the mixing 197AuNP_CD4 with SH‐PEG_2000_‐NH_2_ or SH‐PEG_2000_‐COOH resulted in aggregation. 197AuNP_CD4‐PEG_2000_‐OH can clearly distinguish the CD4^+^/^−^ T cells populations while the mean intensity of SB is 7596 and the mean intensity of NSB is less than 10 (Figure 2B, the blue numeric labels denote mean signal intensity). Unmodified 197AuNP_CD4 resulted much higher mean intensity of NSB (85.7, Figure 2A). 197AuNP_CD4‐PEG_2000_‐NH_2_ and 197AuNP_CD4‐PEG_2000_‐COOH can also distinguish the CD4^+^/^−^ T cells populations, however, the dot plots of CD4^+^ T cells populations were elongated, and the mean intensity was relatively different with median intensity (Figure 2C,D, the red numeric labels denote mean signal intensity and the black numeric labels denote mean signal intensity). This result was contributed to the non‐uniform particle size of aggregated 197AuNP_CD4‐PEG_2000_‐NH_2_ and 197AuNP_CD4‐PEG_2000_‐COOH. From the dot plots of ungated populations (Figure 2E–H) we can see that more AuNP aggregates were observed in the 197AuNP_CD4‐PEG_2000_‐NH_2_ (36%) and 197AuNP_CD4‐PEG_2000_‐COOH (9%) cases. The main reason is that positively charged amino groups induce the serious aggregation of negatively charged gold particles. The uneven charge distribution across the antibody surface may also induce partial aggregation of gold nanoparticles modified by hydroxyl and carboxyl functionalized PEG. Moreover, due to the large size of aggregates, even 193 Ir positive signal was observed in the AuNP aggregates gates, which was used to identify cell nucleus. These aggregates showed no CD45 or CD3 signals, indicating that aggregates could not be mistaken for cells or nuclei (Figure S7). Over all, hydroxyl functionalized PEG was more suitable to modify 197AuNP_CD4.

With this information in hand, we investigated the influence of PEG chain lengths to the NSB lowering efficiency. 197AuNP_CD4 were first modified with SH‐PEG_1000_‐OH and SH‐PEG_5000_‐OH, and then used to stain PBMCs, respectively. The CD4^+^/^−^ T cell populations were gated in Figure S8. 197AuNP_CD4‐PEG_1000_‐OH afforded similar SB intensity compared with that of 197AuNP_CD4‐PEG_2000_‐OH, while the NSB signal was much higher. This result might be due to that shorter PEG cannot provide enough ethylene glycol units to form an efficient brush structure on the surface of AuNP to induce a thermodynamic osmotic stress when approaching to cytoplasmic membrane. In contrast, 197AuNP_CD4‐PEG_5000_‐OH represented both lower SB and NSB signals. This observation demonstrated that long PEG chain facilitates the protein adsorption resistance of 197AuNP_CD4. However, the length of PEG_5000_ is about 30 nm, which is about three times longer than that of IgG (usually ∼14 nm). The long PEG chain might resist the contact between 197AuNP_CD4 and cells, resulting in low SB signals. The chain of PEG_2000_ is about 12 nm and would not shield the antigen‐binding sites of antibodies, resulting in high SB signals. To pursue the sensitivity limitation of AuNP‐based metal tags, higher doses of 197AuNP_CD4‐PEG_2000_‐OH were used to stain PBMCs. As shown in Figure 2I,J and Figure S9, when the doses increased to 272 and 544 K NPs/cell, the mean intensity could reach 11137 and 14035, respectively. Although the mean intensity of NSB were 16.5 and 28.0, which was higher than 10, the median intensity was as low as 3.1 and 6.1. Usually, metal tags can be considered to be successful when the NSB signal was lower than 10. The signal difference between mean and median intensity is contributed to the ultra‐high Au atom content per AuNP. Although 197AuNP_CD4‐PEG_2000_‐OH was only bind to a fraction of the CD4^−^ T cells, a high overall signal intensity was observed. Further increasing of 197AuNP_CD4‐PEG_2000_‐OH would not result higher signal intensity, indicating the reach of adsorption saturation (Figure 2K). As shown in Figure 2I, under mean intensity standard, 197AuNP_CD4‐PEG_2000_‐OH showed a 25‐fold signal amplification while the SB/NSB ratio was 938. Under median intensity standard, 197AuNP_CD4‐PEG_2000_‐OH showed a 46‐fold signal amplification while the SB/NSB ratio was 477, both of which were higher than that of other NPs based metal tags in the direct detection of cell biomarkers.

### 2.2 Preparation and Characterization of PtNP_Abs

**FIGURE 3:**
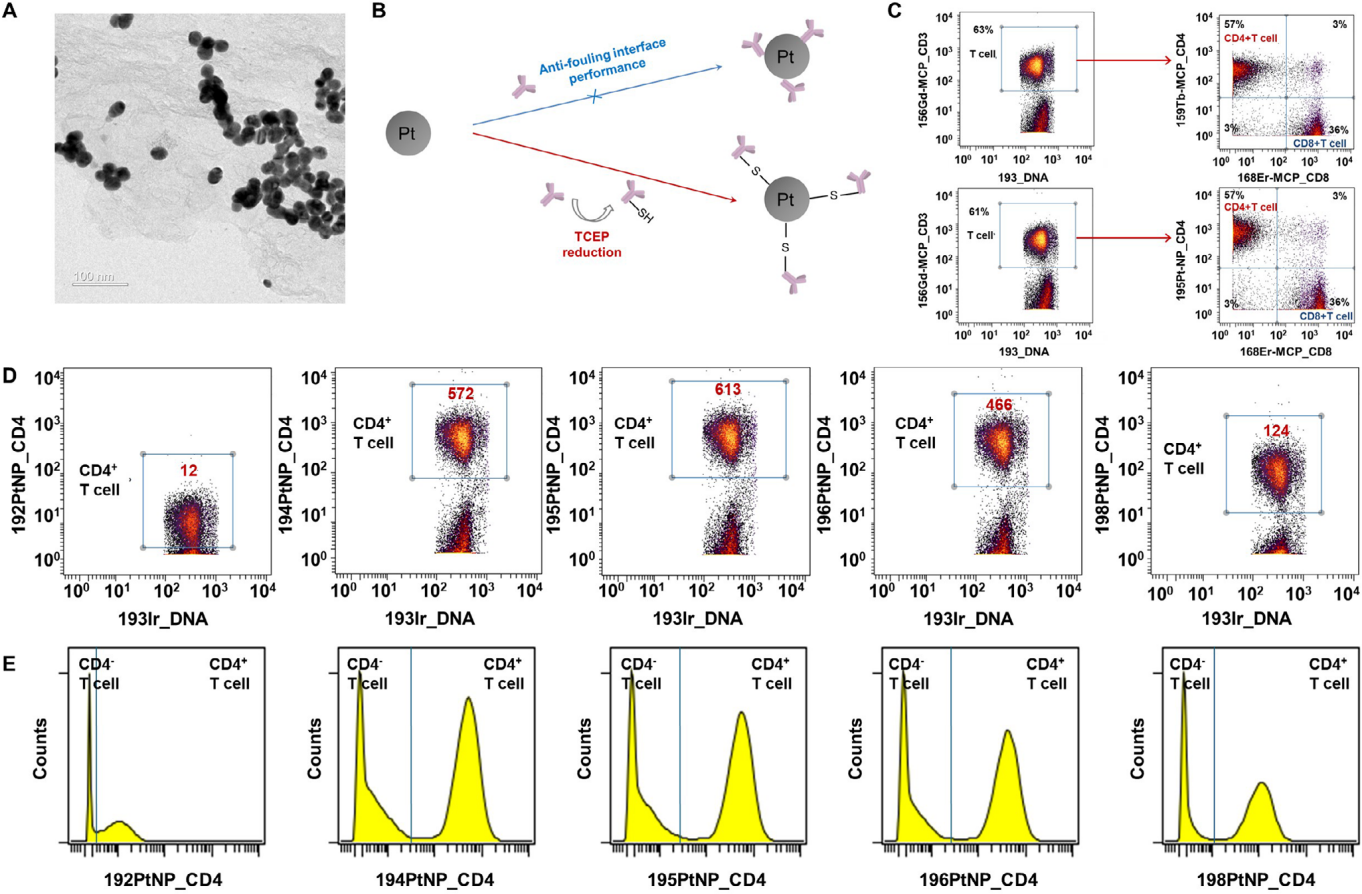
(A) TEM image of PtNP; (B) Conjugation strategy of PtNP and antibody; (C) Dot plots of PBMCs stained by 156Gd‐MCP_CD3, 159Tb‐MCP_CD4, 146Nd‐MCP_CD8 (top) and 156Gd‐MCP_CD3, 195PtNP_CD4, 146Nd‐MCP_CD8 (bottom); (D) Dot plots and (E) histograms of 192Pt, 194Pt, 195Pt, 196Pt and 198Pt‐NPs_CD4 stained T cells.

The PtNP were also synthesized via the simple reduction following a modified reported literature [42]. The resulted product showed spherical morphology with size of about 20 nm as shown in the TEM image (Figure 3A). Due to the excellent anti‐fouling interface performance, the simple mixing of PtNP with anti‐CD4 antibody failed to generate PtNP_CD4 conjugation. The treatment of antibody with tris(2‐carboxyethyl)phosphine (TCEP) will generate thiol groups, which can bind to the surface of PtNP firmly (Figure 3B). The PtNP_CD4 can discriminate CD4^+^ T cells accurately with twofold higher sensitivity (based on 195Pt) and comparable specificity compared with the commercial MCP benchmark as shown in Figure 3C. Pt possesses 5 stable isotopes (192, 194, 195, 196, and 198) with 4 of them being commercially available. As shown in Figure 3D,E, the 192Pt channel can hardly distinguish CD4^+^ T cells population due to the extremely low natural abundance (0.78%). The other 4 channels performed well in cell clustering with SB mean intensity consistent with natural abundances. When the dose of PtNP_CD4 increased from 34 to 272 K NPs/cell, the 195Pt mean intensity of SB increased consequently (Figure S10). When the dose was 68K NPs/cell, the 195Pt mean intensity of NSB was below 10. At this dose, the PtNP_CD4 exhibited 3.6‐fold signal enhancement. Considering the natural abundance of 195Pt is 33.77%, the equivalent mean intensity of 195Pt (100%) can be calculated to 3076 (10.8‐fold higher than MCP tag). The corresponding SB/NSB signal ratio was 150, which was much lower than that of 197AuNP_CD4‐PEG_2000_‐OH due to the lack of PEG modification.

### 2.3 Compatibility with MCP Tags Evaluation

As the commercial MCP metal tags can afford about 40 detection channels and were widely used in all the single cell analysis involving MC, a new metal tag must prove its compatibility with MCP reagents. Here, 197AuNP_CD4‐PEG_2000_‐OH and PtNP_CD4 were used to co‐stain PBMC with 19 MCP metal tags conjugated antibodies, while 159‐MCP_CD4 was used as control. Visual stochastic network embedding (viSNE) was used to analyze the results. From the t‐distributed stochastic neighbor embedding (t‐SNE) contour plots (Figure S11) we can see the introduction of 197AuNP_CD4‐PEG_2000_‐OH and PtNP_CD4 did not interfere with all the rare earth isotope channels and the protein expressions intensity and subgroups gating of PBMCs showed no significant difference. The coefficient of variation (CV) of 197AuNP_CD4‐PEG_2000_‐OH was then tested to evaluate its reproducibility. 10 same PBMC samples were stained with 156Gd‐MCP_CD3, 197AuNP_CD4‐PEG_2000_‐OH, 146Nd‐MCP_CD8, and the population distributions (%) and mean intensity of gated cell subgroups were summarized in Table S1 and Figure S12. The population distributions and mean intensity of MCP and 197AuNP_CD4‐PEG_2000_‐OH gated cell subgroups represent similar CV (∼ 2% for population distributions), demonstrating that noble metal NP based metal tags can be a qualified supplement to the existing commercial MCP metal tags. The 197AuNP_CD4‐PEG_2000_‐OH performance maintained excellent quality throughout the one‐month period (Figure S13). Even after one‐year storage, 197AuNP_CD4‐PEG_2000_‐OH maintained its capacity to distinguish CD4^+^/CD4^−^ T cells, despite a reduction in mean signal intensity to approximately 60%, indicating superior colloidal stability.

### 2.4 Detection and Visualization of Low‐Level CD25 Expression

**FIGURE 4:**
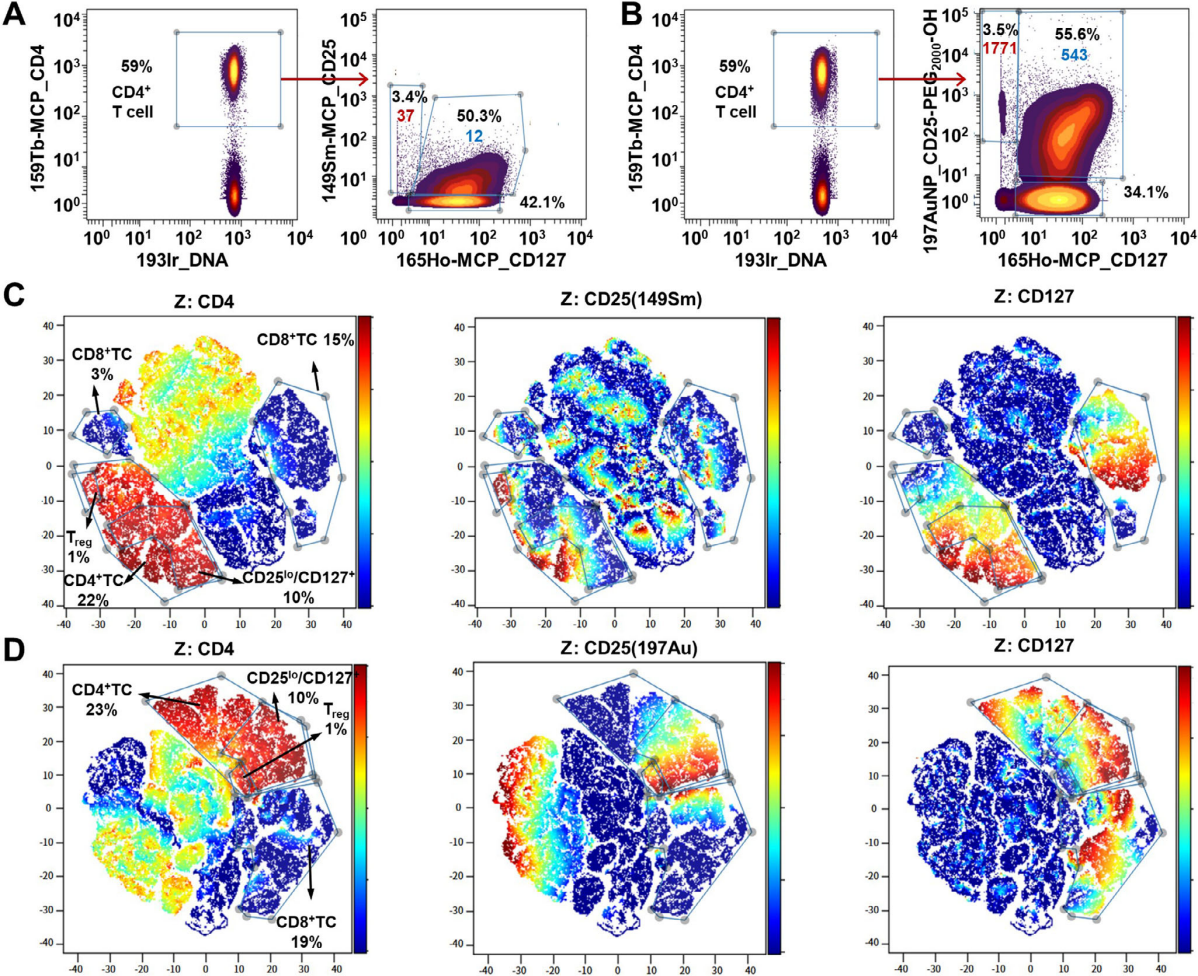
The dot plots of fresh PBMCs stained by 165Ho‐MCP_CD127 and (A) 149Sm‐MCP_CD25 and (B) 197AuNP_CD25‐PEG_2000_‐OH, the red and blue numeric labels denote mean signal intensity; Comparison of CD25 detection in live PBMC by (C) 149Sm‐MCP_CD25 and (D) 197AuNP_CD25‐PEG_2000_‐OH in a viSNE analysis.

The low‐abundant biomarker detection ability of AuNP based metal tag was then tested. CD25 was selected as the target molecule, which was reported to be detected by 40 nm Ag NP with ∼3‐fold signal enhancement [29]. Two fresh PBMC samples were stained with 89Y‐MCP_CD45, 156Gd‐MCP_CD3, 159Tb‐MCP_CD4, 146Nd‐MCP_CD8, 165Ho‐MCP_CD127, and 149Sm‐MCP_CD25/197AuNP_CD25‐PEG_2000_‐OH, respectively. As shown in Figure 4A,B, with 48‐fold (1771/37) signal enhancement, 197AuNP_CD25‐PEG_2000_‐OH can discriminate CD25^hi^/CD127^−^ regulatory T cells (Treg) more distinctly than 149Sm‐MCP_CD25. To the best of our knowledge, 197AuNP_CD25‐PEG_2000_‐OH represent the highest signal amplification in the direct detection of cell biomarkers using NPs based metal tags. More importantly, 149Sm‐MCP_CD25 demonstrates limited efficacy in separation of CD25^lo^/CD127^+^ cells from the CD25^−^/CD127^lo^ population within CD4^+^ T cells in biaxial dot plots, which can be easily discriminated by highly sensitive 197AuNP_CD25‐PEG_2000_‐OH with 45‐fold (543/12) signal enhancement. In computational data analysis using the dimensionality reduction tool viSNE, both 149Sm‐MCP_CD25 and 197AuNP_CD25‐PEG_2000_‐OH can clearly mark Tregs (Figure 4C,D). However, only CD25 detection by 197AuNP_CD25‐PEG_2000_‐OH enable the mediation of the clustering of CD25^hi^/^lo^/^−^ CD4^+^ T cells as adjacent cells respectively in a viSNE plot. With all the information above, we compared our mass tag with other reported nanoparticle typed mass tags from different perspectives, and the result was summarized in Table S2. In conclusion, our noble metal nanoparticle‐based mass tags were superior to most of the published mass tags.

### 2.5 A Multiplexed SMC Assay with 197AuNP_CD4‐PEG_2000_‐OH and PtNP_CD8

**FIGURE 5:**
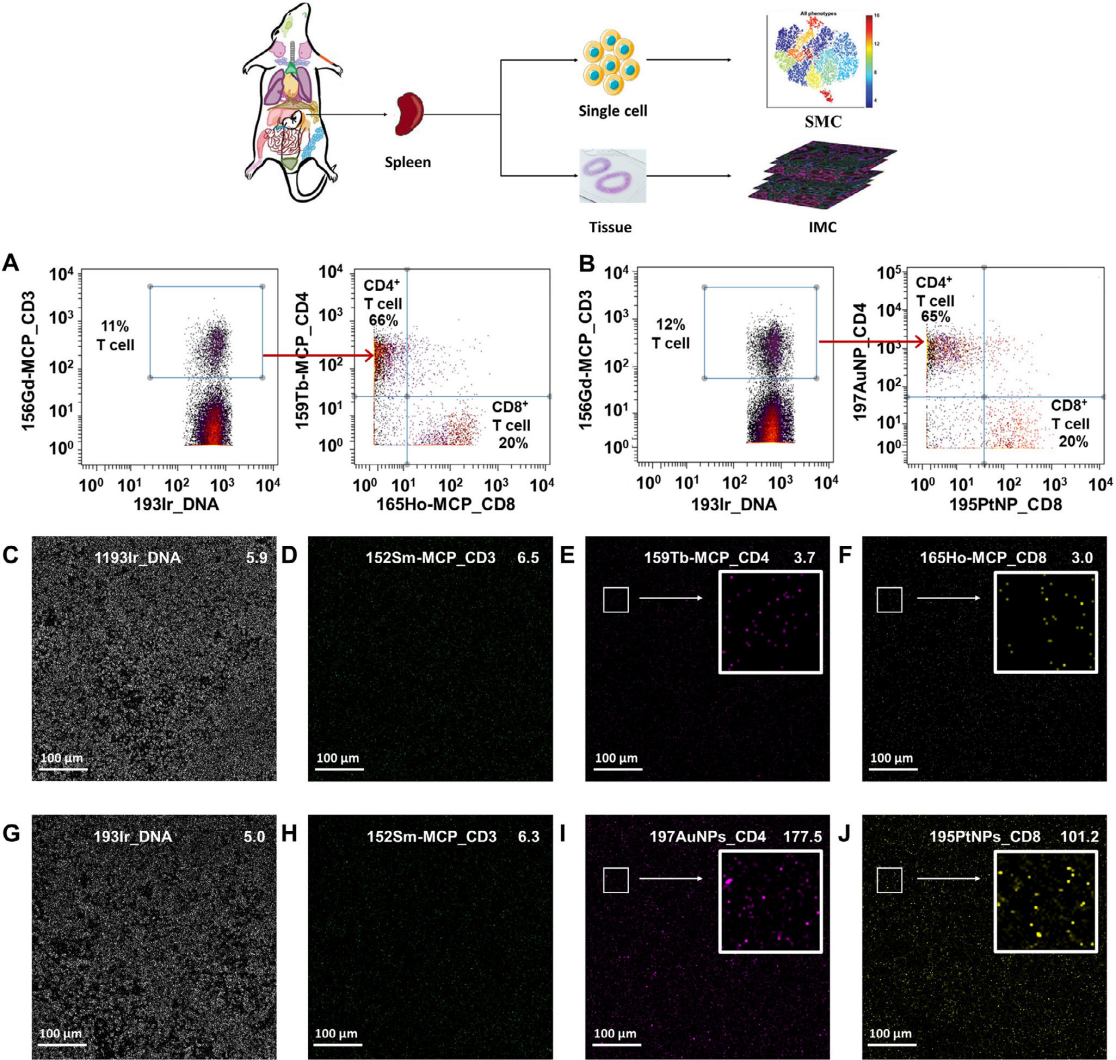
Dot plots of CD4^+^/CD8^+^ T cells stained by (A) 159Tb‐MCP_CD4 and 165Ho‐MCP_CD8 and (B) 197AuNP_CD4‐PEG_2000_‐OH and 195PtNP_CD4; IMC images for ROI 1. white (C), lime (D), magenta (E) and yellow (F)are assigned to 193Ir_DNA, 152Sm‐MCP_CD3, 159Tb‐MCP_CD4 and 165Ho‐MCP_CD8, respectively; white (G), lime (H), magenta (I) and yellow (J) is assigned to 193Ir_DNA, 152Sm‐MCP_CD3, are 197AuNP_CD4‐PEG_2000_‐OH and 195PtNP_CD8, respectively. Scale bar shown on the lower left conner is 100 µm. The max threshold value is shown on the upper right corner.

Tissue samples exhibit inherently greater complexity than cell samples, and certain metal tags optimized for single‐cell staining are ineffective in tissue staining [43, 44]. Till now, a limited number of NPs based metal tags have been incorporated into IMC experiments [25]. Here, AuNP and PtNP were conjugated to anti‐mouse CD4 and CD8, respectively, to co‐stain mouse spleen tissue (labeled as tissue 1) with 152Sm‐MCP_CD3 and an Ir intercalator. 152Sm‐MCP_CD3, 159Tb‐MCP_CD4, 165Ho‐MCP_CD8 and an Ir intercalator were used to stain another mouse spleen tissue (labeled as tissue 2) as control. These panels were proven to be efficient in distinguish mouse spleen cells as shown in Figure 5A,B. The image of mouse spleen tissues is shown in Figure S14. Three regions of interest (ROIs) were selected to evaluate the staining performance. The ROIs were detected by IMC through laser ablation in 1 µm^2^ pixels and the representative images from ROI 1, ROI 2, and ROI 3 are shown in Figure 5, Figures S15 and S16. The max threshold value for each image, displayed in the upper right corner, corresponds to the 98th percentile of per‐pixel metal counts derived from the pixel containing the highest metal concentration. Any pixel with a metal count higher than the max threshold is assigned a color (white for 193Ir, lime for 152Sm, magenta for 159 Tb/197Au and yellow for 165Ho/195Pt). This value offers a relative quantitative metric for evaluate the brightness of the IMC image. In general, a higher max threshold value renders enhanced image contrast by expanding the grayscale dynamic range of metal counts detected via IMC. As shown in Figure 5D,H, the images for 152Sm‐MCP_CD3 stained tissue 1 and 2 represent similar max threshold value at ∼ 6, resulting lime color at an equivalent brightness level. In contrast, when 197AuNP_CD4‐PEG_2000_‐OH and PtNP_CD8 were used to stain tissue 2, both channels represent much higher max threshold values (47‐fold higher for AuNP and 33‐fold higher for PtNP), which is corresponding with the SMC results. The brightness of tissue 2 was also much higher than that of tissue 1 even under higher max threshold, demonstrating promising IMC metal tags. As tissue samples are more likely to absorb nanoparticles than cell suspension samples, AuNP/PtNP and AuNP‐PEG_2000_‐OH /PtNP were used to stain the tissues as control. As shown in Figure S17, the unmodified AuNP showed much high signals than 197AuNP_CD4‐PEG_2000_‐OH while AuNP‐PEG_2000_‐OH showed lower signals. This result indicated that the PEG modification can significantly reduce the NSB level and 197AuNP_CD4‐PEG_2000_‐OH indeed combine to tissue via antigen‐antibody specific binding.

### 2.6 AML‐Associated T Cell Remodeling: Distinct Subpopulations and Immune Checkpoint Dysregulation

**FIGURE 6:**
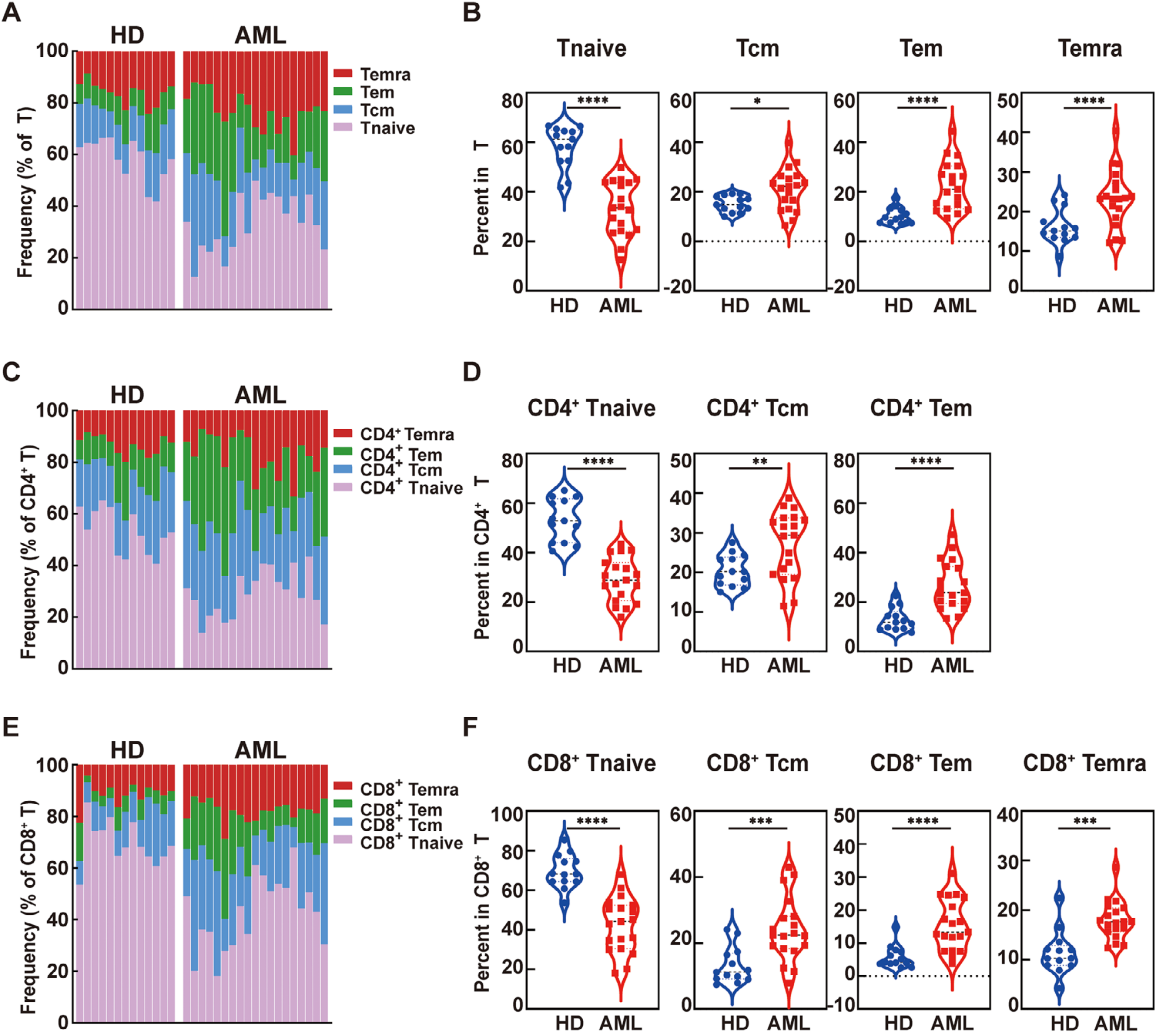
Phenotypic Profiling of T Cell Differentiation in AML Patients (A) Representative high‐dimensional data gated on T according to CD45RA and CCR7 (B) Proportion of naïve (CD45RA^+^CCR7^+^, Tnaive), central memory (CD45RA^−^CCR7^+^, Tcm), effector memory (CD45RA^−^CCR7^−^, Tem) T cells and terminally differentiated effector memory (CD45RA^+^CCR7^−^, Temra) over the total T cell subsets in HD (N = 13), and in patients AML (N = 19) (C) CD4^+^ T cell subsets according to CD45RA and CCR7. (D) Proportion of Tnaive, Tcm, and Tem among CD4^+^ T cells in HD and AML (E) CD8^+^ T cell subsets according to CD45RA and CCR7. (F) Proportion of Tnaive, Tcm, Tem, and Temra among CD8^+^ T cells in HD and AML.

**FIGURE 7:**
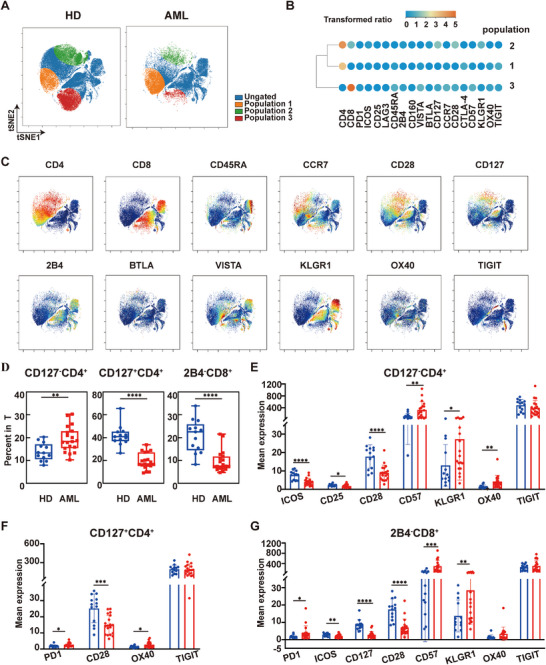
Immune checkpoint changes in T cells (A) Overlaid viSNE maps colored by indicated cell populations; (B) Heatmap displaying the normalized median expression of indicated biomarkers across three populations; (C) ViSNE maps colored by the expression of indicated markers; (D) Bar plots showing the significantly altered frequencies of the indicated cell populations; (E‐G) Bar plots depicting the mean expression of indicated markers in (E) CD127^−^CD4^+^ T, (F) CD127^−^CD4^+^ T, and (G) 2B4^−^CD8^+^ T.

After the detailed evaluating of AuNP and PtNP‐based metal tags, they are used to conjugate antibodies targeting low‐abundant biomarkers for immune checkpoint profiling in AML. TIGIT and OX40 were selected to conjugate with AuNP and PtNP as MCP tags (154Sm‐MCP_TIGIT and 158Gd‐MCP_OX40) show low sensitivity in the MC detection of these two biomarkers of PBMC (Figure S18). 24 AML related biomarkers were examined in 13 HD and 19 AML samples. The demographic characteristics of all participants are presented in Table S3 and the full antibody panel is described in Table S4. We conducted immunophenotypic analyses of T cells from AML patients to characterize their differentiation status compared to HD. The gating strategy was shown in Figure S19. The results revealed a marked skewing of T cell differentiation in AML, with significantly reduced proportions of naive T cells (Tnaive) and increased populations of mature subsets (Tcm, Tem, and particularly elevated Temra in T and CD8^+^ T cells) across all T cell compartments when compared to HD (Figure 6). We employed viSNE to analyze T cell distribution in both 9 HD and 9 AML, all of whom were randomly selected from the total participant cohort, identifying three significantly distinct T cell subpopulations (Figure 7A). Comparative to HD, AML exhibited a substantial increase in Population 1 frequency, but significant reductions in Populations 2 and 3 (Figure S20A,D,G). To functionally characterize these subpopulations, we visualized the median‐normalized expression profiles of 12 T cell immunophenotypic and immune checkpoint markers as a heatmap across the three subpopulations (Figure 7B). Concurrently, we systematically mapped the expression of these biomarkers onto the viSNE plots, revealing their expression levels and spatial distribution patterns (Figure 7C). Integrating the above analysis with biomarker mean expression levels and statistical assessments, we identified the most representative subsets: CD127^−^CD4^+^, CD127^+^CD4^+^ and 2B4^−^CD8^+^ T cells (Figure S20). CD127 is a surface marker for memory T cells, which promotes the homeostatic proliferation of T cells. CD127^+^CD4^+^ T cell subsets (such as Th1, Th2, Th17, etc.) can produce various cytokines (such as IFN‐γ, IL‐2, IL‐4, IL‐17, etc.) and play crucial roles in anti‐tumor immunity and mediating inflammatory responses [45]. Moreover, 2B4^−^CD8^+^ T cells possess a stronger capacity for self‐renewal, proliferative differentiation, formation and maintenance of long‐term immune memory, and the ability to proliferate and differentiate into new effector cells [46]. They serve as a reservoir for immune responses and are a key functional subset for reversing immune exhaustion and restarting immune responses. Comparative analysis revealed significantly reduced frequencies of CD127^+^CD4^+^ and 2B4^−^CD8^+^ T cell subsets in AML compared to HD, suggesting memory T‐cell exhaustion contributes to tumor immune escape in AML.

Furthermore, an expanded sample cohort was utilized to investigate the differences in the CD127^−^CD4^+^, CD127^+^CD4^+^, and 2B4^−^CD8^+^ T cell subpopulations between 13 HD and 19 AML. Results demonstrated that compared with HD, the proportion of CD127^−^CD4^+^ T cells was significantly elevated in AML, whereas the proportions of CD127^+^CD4^+^ and 2B4^−^CD8^+^ T cells were significantly reduced (Figure 7D). These observations are consistent with the aforementioned analysis. Next, we systematically analyzed the expression profiles of immune checkpoint proteins across three subsets. In CD127^−^CD4^+^ T cells, AML exhibited significant upregulation of KLGR1, OX40, and CD57, alongside downregulation of ICOS, CD28, and CD25 relative to HD (Figure 7E). Consistently, in CD127^+^CD4^+^ T cells, PD1 and OX40 were upregulated, while CD28 were downregulated in AML (Figure 7F). The 2B4^−^CD8^+^ T cells showed the most extensive alterations, with AML displaying upregulation of PD1, KLGR1, and CD57, coupled with downregulation of ICOS, CD28, and CD127 compared to HD (Figure 7G). Our detection of immune checkpoint proteins [47, 48, 49, 50] revealed a complex pattern of immune checkpoint modulation in AML samples across three T‐cell subsets. While T‐cell activation‐stimulating factors such as CD28 and ICOS were significantly downregulated, activation‐inhibiting factors such as TIGIT exhibited upregulation. This suggests that the inhibition of T‐cell function may be a cause for immune escape of AML. However, some T‐cell activation‐stimulating factors like OX40 were expressed at higher levels in AML samples. This indicates that the homeostasis of T cells is disrupted in the AML microenvironment, collectively contributing to immune escape of leukemia cells. These findings may provide new targets and intervention strategies for the diagnosis and treatment of AML. The successful detection of TIGIT and OX40 using AuNP and PtNP tags also demonstrates that noble metal NP‐based tags show promising potential for high‐dimensional single‐cell immune profiling via MC and the detection of other low‐abundance cell surface biomarkers.

## 3 Conclusion

In this work, for the first time, we demonstrate the feasibility and superiority of antibody‐functionalized noble metal nanoparticles as metal tags for biomarker detection with single‐cell resolution in both SMC and IMC. Compared with other NPs based metal tags, the noble metal NPs based metal tags exhibited much simpler synthetic process and higher isotopic utilization. Even extremely low‐dose AuNP (∼34 NPs/cell) achieved comparable sensitivity to the commercial MCP tags, while high doses enabled up to 48‐fold signal amplification in the direct detection of cell surface biomarkers. Integrated detection of TIGIT/OX40 via Au/PtNP tags and 22 MCP‐tagged biomarkers revealed three novel T‐cell subpopulations in HD/AML cohorts, indicating the disruption of T‐cell homeostasis within the AML microenvironment collectively facilitates immune escape of leukemia cells. This study provides a simple metal label strategy for SMC and IMC multiparameter and sensitive single‐cell biomarker dual detection and demonstrate its promising potential for practical biomedical applications.

## Author Contributions

Zhizhou Liu, Yu Yang, and Lingfei Qiu contributed equally to this work. P. Bai performed conceptualization. Z. Liu, Yu Yang, Lingfei Qiu, Yu Yang, and Juan Yue performed the methodology. Zhizhou Liu, Yu Yang, Lingfei Qiu, and Jingrui Li performed the Investigation. Zhizhou Liu, Yu Yang, and Lingfei Qiu wrote the original draft. Yinghui Li, Yingdai Gao, and Pengli Bai reviewed and edited the original draft.

## Conflicts of Interest

The authors declare no conflicts of interest.

## Ethics Approval Statement

All primary cells followed the Declaration of Helsinki and were approved by the Ethics Review Board of the Institute of Hematology and Blood Diseases Hospital, Chinese Academy of Medical Sciences. Informed written consent was obtained from all donors according to humanitas ethical committee regulations from the Institute of Hematology and Blood Diseases Hospital (ethical review approval No: KT2020024‐EC‐2). Animal experimental protocols received approval NO. IHCAMS‐DWLL‐NSFC2024010‐1 from the Animal Care and Use Committee of State Key Laboratory of Experimental Hematology, Institute of Hematology and Blood Diseases Hospital. All mouse experimental procedures were performed in accordance with the Regulations for the Administration of Affairs Concerning Experimental Animals approved by the State Council of the People's Republic of China.

## Supporting information




**Supporting File**: advs74529‐sup‐0001‐SuppMat.docx.

## Data Availability

The data that support the findings of this study are available from the corresponding author upon reasonable request.
