# Supplementary material for: High‐Sensitive Mass Cytometry Metal Tags Based on Noble Metal Nanoparticles Assisting Acute Myeloid Leukemia Immune Checkpoint Profiling
Source: Adv Sci (Weinh). 2026 Mar 2;13(25):e74529. doi: 10.1002/advs.74529 (PMC13137793; doi:10.1002/advs.74529)
Supplement: Supplementary file 1 — Supporting File: advs74529‐sup‐0001‐SuppMat.docx. [file ADVS-13-e74529-s001.docx]

High-sensitive mass cytometry metal tags based on noble metal nanoparticles assisting acute myeloid leukemia immune checkpoint profiling

*Zhizhou Liu ^a, b#^, Yu Yang ^c#^, Lingfei Qiu ^a,^ ^d#^, Yi Yang ^a^, Jingrui Li ^a^, Juan Yue ^a^, Yinghui Li ^e*^, Yingdai Gao ^e^, Pengli Bai ^a*^*

a Suzhou Institute of Biomedical Engineering and Technology, Chinese Academy of Sciences, Suzhou, Jiangsu 215163, China

b Tianjin Guoke Medical Engineering and Technology Development Co., Ltd, Tianjin 300300, China

c School of Precision Instrument and Opto-Electronics Engineering, Tianjin University, Tianjin 300072, China

d Department of Chemistry, College of Sciences, Shanghai University, Shanghai 200444, China

e State Key Laboratory of Experimental Hematology, Institute of Hematology and Blood Diseases Hospital, Chinese Academy of Medical Sciences and Peking Union Medical College, Tianjin 300020, China.

# These authors contributed equally.

*Corresponding author: Pengli Bai, [baipl@sibet.ac.cn](mailto:baipl@sibet.ac.cn)

Yinghui Li, liyinghui@ihcams.ac.cn

Table of Contents

[Experimental Section 3](#_Toc218684711)

[Ethical Issues 3](#_Toc218684712)

[Materials and Reagents 3](#_Toc218684713)

[Apparatus and Characterization 4](#_Toc218684714)

[Synthesis of Au Nanoparticles (AuNPs) 5](#_Toc218684715)

[Synthesis of Pt Nanoparticles (PtNPs) 5](#_Toc218684716)

[AuNP-Antibody Conjugation and calculation 5](#_Toc218684717)

[PtNP-Antibody Conjugation 6](#_Toc218684718)

[Cell Staining Experiments with PBMCs 6](#_Toc218684719)

[Preparation and Staining of Tissue Sections 7](#_Toc218684720)

[Human Specimens 8](#_Toc218684721)

[Sample Processing 8](#_Toc218684722)

[Antibody Panels and Staining 9](#_Toc218684723)

[Mass Cytometry and Data Acquisition 9](#_Toc218684724)

[ViSNE Settings 10](#_Toc218684725)

[Statistical Analysis 10](#_Toc218684726)

[Supporting Figures 11](#_Toc218684727)

[Supporting Tables 26](#_Toc218684728)

[References 30](#_Toc218684729)

# Experimental Section

## Ethical Issues

All primary cells followed the Declaration of Helsinki and were approved by the Ethics Review Board of the Institute of Hematology and Blood Diseases Hospital, Chinese Academy of Medical Sciences. Informed written consent was obtained from all donors according to humanitas ethical committee regulations from the Institute of Hematology and Blood Diseases Hospital (ethical review approval No: KT2020024-EC-2). Animal experimental protocols received approval NO. IHCAMS-DWLL-NSFC2024010-1 from the Animal Care and Use Committee of State Key Laboratory of Experimental Hematology, Institute of Hematology and Blood Diseases Hospital. All mouse experimental procedures were performed in accordance with the Regulations for the Administration of Affairs Concerning Experimental Animals approved by the State Council of the People’s Republic of China.

## Materials and Reagents

HAuCl_4_·3H_2_O and H_2_PtCl_6_·3H_2_O were purchased from Adamas-beta®. Sodium citrate, citric acid, heparin, and sodium borohydride were purchased from Tansoole Co. Ltd. M-xylene and TCEP were obtained from Sigma-Aldrich. All PEGs used were purchased from Peking PEG. Antibodies were obtained from BioLegend or BD summarized in Table S4 Ir intercalator, Multi-Metal Maxpar ® Kit, Maxpar ® Fix and Perm Buffer, Maxpar ® Cell Staining Buffer (CSB) and EQ Four Element Calibration Beads were purchased from Fluidigm. MCP tags were prepared using Multi-Metal Maxpar ® Kit with different antibodies according to the Fluidigm antibody labeling protocol. All chemicals listed were used as received without further purification. Normal mouse spleen tissues were supplied by SPF (Suzhou) Biotechnology Co., Ltd. PBMCs of HD were isolated from human peripheral blood samples derived from healthy donors who has signed a written informed consent. PBMCs of AML were isolated from human peripheral blood samples derived from AML patients recruited at the Institute of Hematology and Blood Diseases Hospital, Chinese Academy of Medical Sciences & Peking Union Medical College (CAMS & PUMC). The included AML cells were from relapsed/refractory AML patients.

## Apparatus and Characterization

Transmission electron microscopy (TEM) and high resolution energy dispersive X-ray spectroscopy (EDX) were performed on a Tecnai G2 F20 TEM spectroscope. UV/visible titration profiles were carried out with Hitachi U-3900H spectrophotometer. Zeta potential and DLS size were measured on a Malvern Zetasizer. Cell mixture was analyzed by mass cytometry (CyTOF® Helios™ system at Fluidigm Canada, events mode). Data were collected in the dual counting mode and processed with Cytobank software. Imaging mass cytometry experiments were run at Standard BioTools in Markham, ON using a Hyperion Imaging system. Data were collected in the dual counting mode and processed with MCD Viewer software.

## Synthesis of Au Nanoparticles (AuNPs)

AuNPs were prepared by reducing HAuCl_4_ with sodium citrate. 50 mL of 1 mM HAuCl_4_ solution was heated to boiling, and then 5 mL of 1% sodium citrate solution was quickly added; the AuNPs were obtained after continuous stirring and refluxing for 15 min.

## Synthesis of Pt Nanoparticles (PtNPs)

PtNPs were synthesized via aqueous reduction of chloroplatinic acid hexahydrate (H_2_PtCl_6_·3H_2_O) in the presence of citrate ions. Typically, A 0.2% (w/v) H_2_PtCl_6_·3H_2_O solution (0.36 mL) was injected into 4.64 mL of deionized water under vigorous stirring. Subsequently, 0.11 mL of an aqueous solution containing 1% (w/v) sodium citrate and 0.05% (w/v) citric acid was added. After 30 s, 0.055 mL of a freshly prepared 0.08% (w/v) sodium borohydride solution containing 1% sodium citrate and 0.05% citric acid was rapidly injected into the reaction system. The colloidal solution was stirring for 10 min.

## AuNP-Antibody Conjugation and calculation

0.5 mL colloidal AuNP solution was centrifuged at 8000 rpm for 30 min and dispersed in 0.5 mL deionized water, to which 20 μL antibody (0.5 mg/mL) was added. The mixture was vortexed for 10s and reacted for 8 h, after which 5 mg of thiol functionalized PEG was added. After another 8 h the mixture was centrifuged at 8000 rpm for 30 min to remove unreacted antibody and PEG. Then the AuNP_Ab-PEG was dispersed in 0.5 mL deionized water and stored at 4 °C. The antibody concentration before and after the conjugation reaction was determined to be 0.543 mg/mL (20 μL) and 0.0104 mg/mL (500 μL) by a Bradford protein assay. The total amount of bound Ab was (0.543 mg/mL × 20 μL - 0.0104 mg/mL × 500 μL)/150 KDa × 6.02 ×10^23^ = 2.3 ×10^13^. The number of AuNP was 0.5 mL × 3.4 × 10^13^/mL = 1.7 × 10^13^. The average number of antibody per AuNP was 2.3 ×10^13^ / 1.7 × 10^13^=1.4.

## PtNP-Antibody Conjugation

20 μg antibody was dissolved in 20 μL R-buffer, and to which 20 μL R-buffer containing 4 mM TCEP was added. The mixture was incubated at 37 °C for 30 min. Meanwhile, 1.5 mL colloidal PtNP solution was centrifuged at 14000 rpm for 30 min and dispersed in 0.75 mL deionized water. The partial reduced antibody was then added to colloidal PtNP solution and reacted for 8 h. The PtNP_Ab was dispersed in 0.4 mL deionized water and stored at 4 °C.

## Cell Staining Experiments with PBMCs

1 million fixed PBMCs were thawed and washed by 5 mL CSB twice at 600 g and the supernatant was removed. The cell pellet collected were resuspend with 50 μL CSB and 5 μL FcR Blocking Solution was added at room temperature. After 10 min, an Ab cocktail (50 µL) was added and vortexed gently. The mixture was combated at room temperature for 45 min. Then the cells were washed by CSB twice to remove unbind antibodies and stained with 1 mL of Ir DNA intercalator (500 µM, 2.5 µL) solution in fix and perm buffer at room temperature for 1 h. Before MC examination, the cells were centrifuged at 600 g for 5 min and the cell pellet was washed by CSB (600 g) once and deionized water (600 g) twice. The cell pellet was set on ice until test. For testing, the cell pellet was dispersed with 10% EQ Four Element Calibration Beads and filtered with a 30 μm Nylon mesh to remove large-size particulate impurities. The cell sample was run on the Helios instrument for data acquisition.

## Preparation and Staining of Tissue Sections

The tissue was stained following the “Imaging Mass Cytometry Staining Protocol for FFPE sections” (Standard BioTools Inc.) containg six steps: 1) Dewaxing; 2) Hydration; 3) Antigen retrieval; 4) Blocking; 5) Antibody staining; 6) Intercalator staining. Typically, the tissue slides were heated for 2 h in an oven preheated at 60 °C to remove the visible wax. 40 mL antigen retrieval solution was loaded in a 50 mL conical tube and heated in a water bath at 96 °C with the cap loosely fitted. The tissues were then dewaxed in fresh M-xylene for 20 min. Then the hydration process was performed using ethanol containing different percentages of water (100%, 95%, 80%, and 70% ethanol) for 5 min each. The slides were washed by water for 5 min in a Coplin jar with gentle shaking. The slides were then incubated in the preheated antigen retrieval solution at 96 °C for 30 minutes with a loose cap. The conical tube was then removed from water bath and cooled to about 70 °C gradually in 10 min. The slides were washed by water and PBS for 10 min respectively in a Coplin jar with gentle shaking. After encircled with a PAP pen, the tissue was blocked by BSA (3% in PBS) for 45 min in a hydration chamber. The BAS solution was removed and Ab cocktail was added. The slides were left in the hydration chamber at 4 °C overnight. Then the slides were washed with Triton X-100 (0.2 % in PBS) for 8 minutes with gentle shaking for 4 times. The tissues were then stained with Ir DNA intercalator solution for 30 minutes are room temperature (RT) in a hydration chamber. Finally, the tissues were washed with water for 5 min with gentle shaking and dried in air for 20 min at RT.

## Human Specimens

Informed consent was obtained from all participants in accordance with the Declaration of Helsinki, including healthy donors (HDs, n=9) and acute myeloid leukemia (AML) patients (n=9). AML patients were recruited at the Institute of Hematology and Blood Diseases Hospital, Chinese Academy of Medical Sciences & Peking Union Medical College (CAMS & PUMC). The study protocols were reviewed and approved by the institutional Ethics Committee. Clinical and demographic characteristics of all participants are presented in Table S3.

## Sample Processing

Whole blood (WB) samples from healthy donors and patients were collected within 4 hours and centrifuged at 3000 × g for 10 minutes to separate and remove plasma. The remaining cells were incubated with Red Blood Cell Lysing Buffer for 15 minutes at RT, followed by centrifugation at 300 × g for 5 minutes to obtain white blood cells (WBCs). The WBCs were then washed once with PBS and resuspended in pre-warmed FBS-free DMEM (Gibco). After washing, cells were stained with cisplatin for 5 minutes at 37 °C to discriminate live/dead cells. Staining was terminated by adding CSB and centrifugation. Supernatants were discarded, and cells were fixed in 1.6% paraformaldehyde solution at RT for 10 minutes. Finally, cells were cryopreserved in DMSO-containing CSB at −80°C after washing.

## Antibody Panels and Staining

Cryopreserved WBCs were thawed in a 37°C water bath for 5 min. For each sample, aliquots of 1-2 million cells were washed sequentially with CSB and CSB plus 400 U/ml heparin. After 10-minute Fc-blocking at RT, cells were stained with surface antibodies (Table S4) for 45 min with 15-min vortex intervals, followed by two CSB washes. Final fixation used Fix/Perm Buffer (Fluidigm) containing 125 nM iridium intercalator for DNA staining and antibody stabilization.

## Mass Cytometry and Data Acquisition

Prior to data acquisition, cells were washed with one CSB wash and two washes in ultra-pure water to eliminate excess intercalator, then resuspended in ultra-pure water containing 10% EQ Four Element Calibration Beads (Fluidigm) for instrument calibration. Approximately 100,000 cell events were acquired on a Helios at an event rate of 200-400 events per second. After data acquisition, EQ beads were removed via fluctuation-correction algorithm, with data exported as .fcs files to Cytobank for further analysis.

## ViSNE Settings

ViSNE clustering was performed using T cell-related antibodies, as detailed in STable S4. All T cell event sampling was conducted for each sample. ArcSinh scale was used and the cofactor was set to 150 in Cytobank. Default Iterations, perplexity and theta were applied to these data.

## Statistical Analysis

Statistical analyses were conducted using GraphPad Prism 9.0. Data are expressed as mean ± standard deviation (SD). Intergroup comparisons were performed using the non-parametric Mann-Whitney U test, with p < 0.05 considered statistically significant.

# Supporting Figures


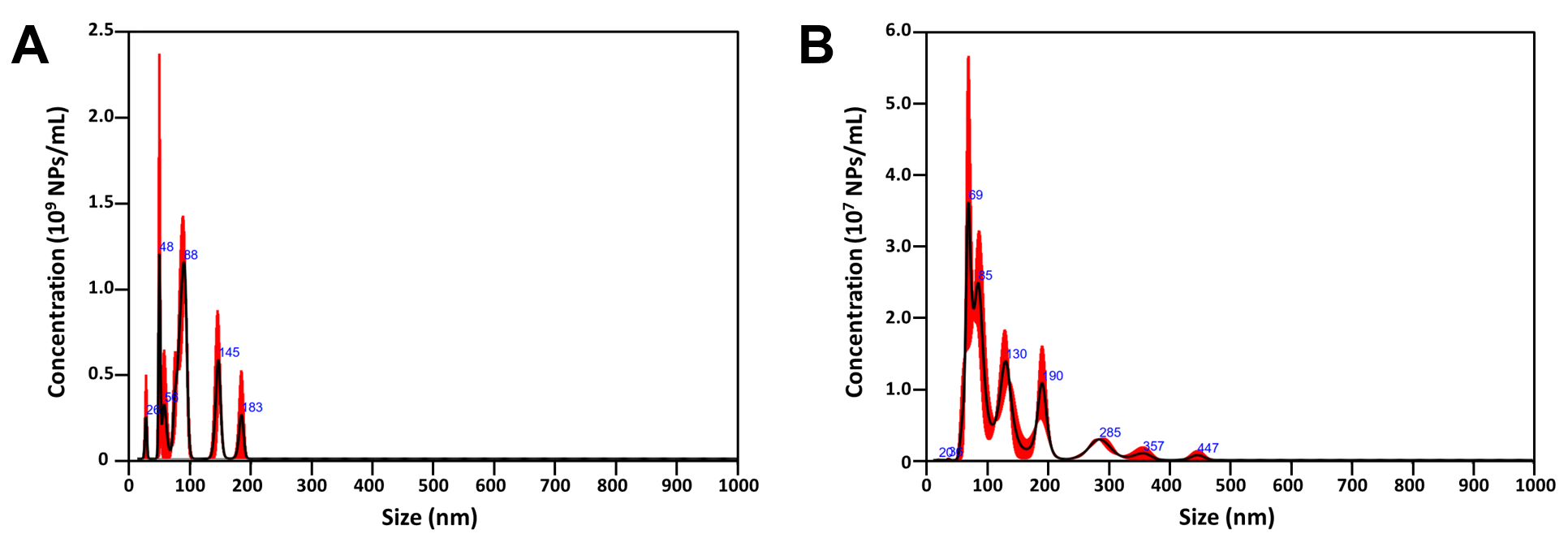


**Figure S1** NP concentration of (A) AuNPs (Dilution 1: 1000) and (B) PtNPs (Dilution 1: 10) investigated by NanoSight.


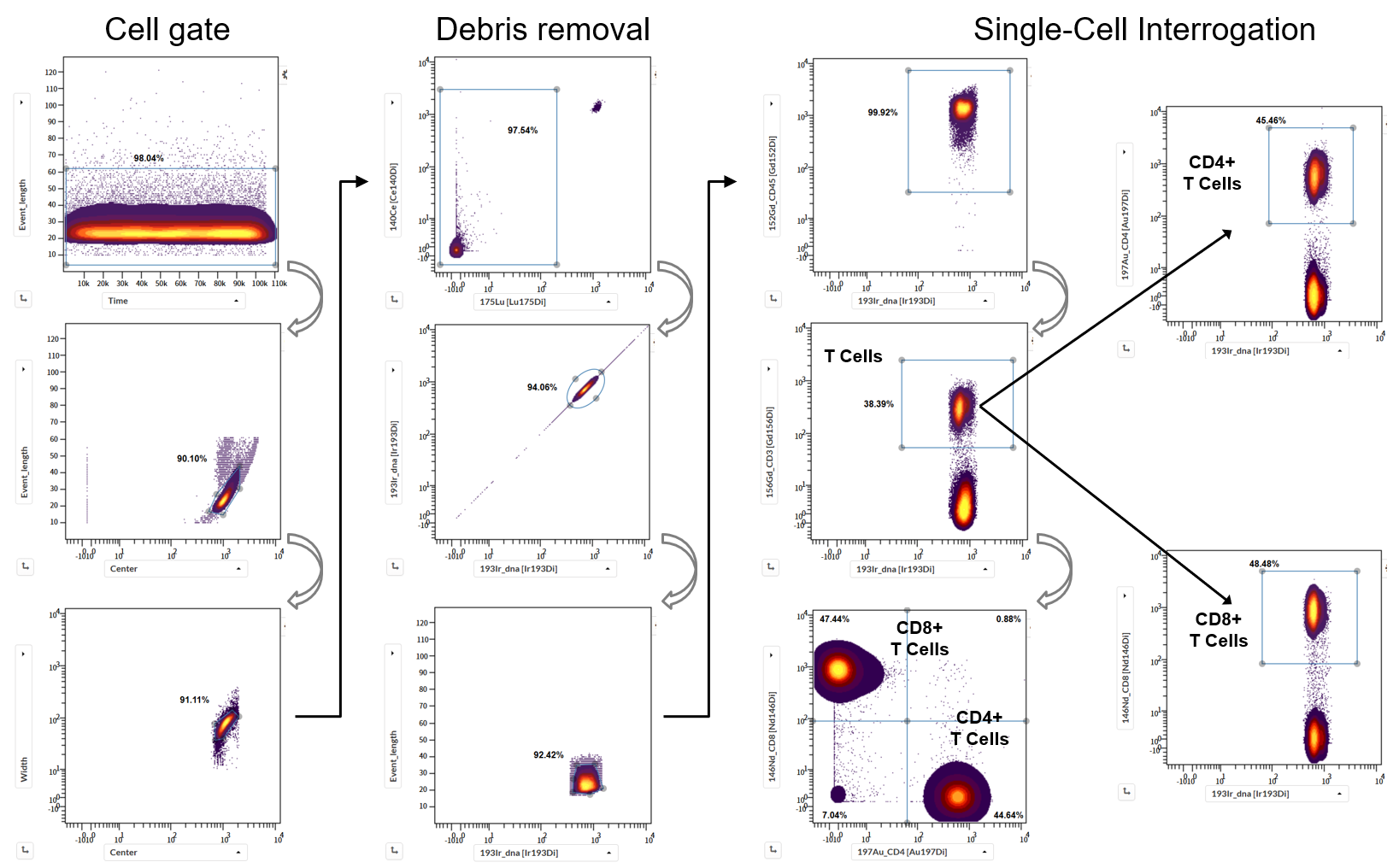


**Figure S2** CyTOF single cell gating strategy.


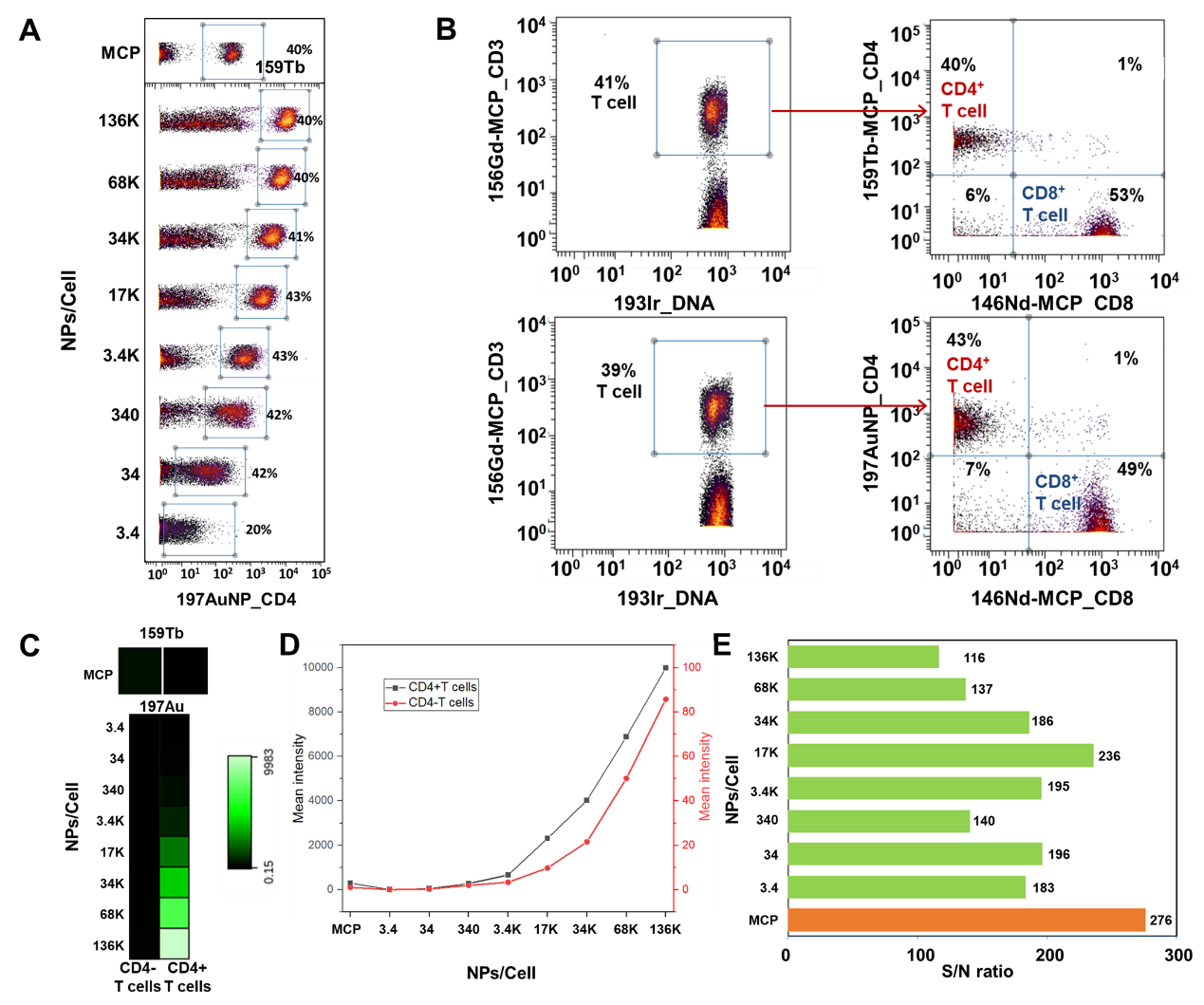


**Figure S3** (A) Dot plots of T cells stained by 159Tb-MCP_CD4 and different dose of 197AuNP_CD4; (B) Dot plots of PBMCs stained by 156Gd-MCP_CD3, 159Tb-MCP_CD4, 146Nd-MCP_CD8 (top) and 156Gd-MCP_CD3, 197AuNP_CD4, 146Nd-MCP_CD8 (bottom); (C) heatmap of T cells stained by 159Tb-MCP_CD4 and different dose of 197AuNP_CD4; (D) The mean intensity of CD4^-^ and CD4^+^ T cells; (E) The SB/NSB ratio.


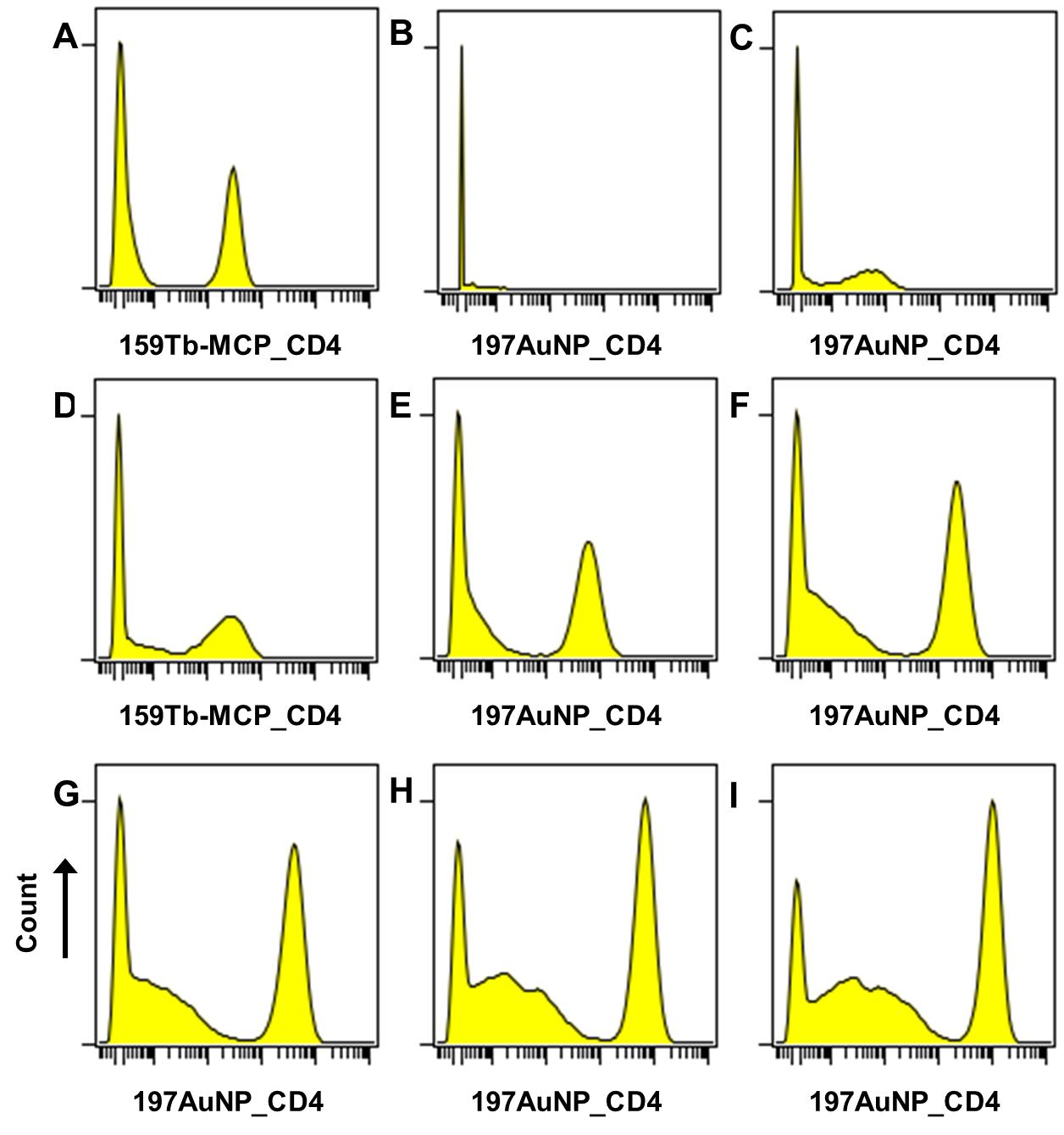


**Figure S4** The histograms of T cells stained by 159Tb-MCP_CD4 (A) and different dose of 197AuNP_CD4 (B-F, 3.4-136K NPs/cell).


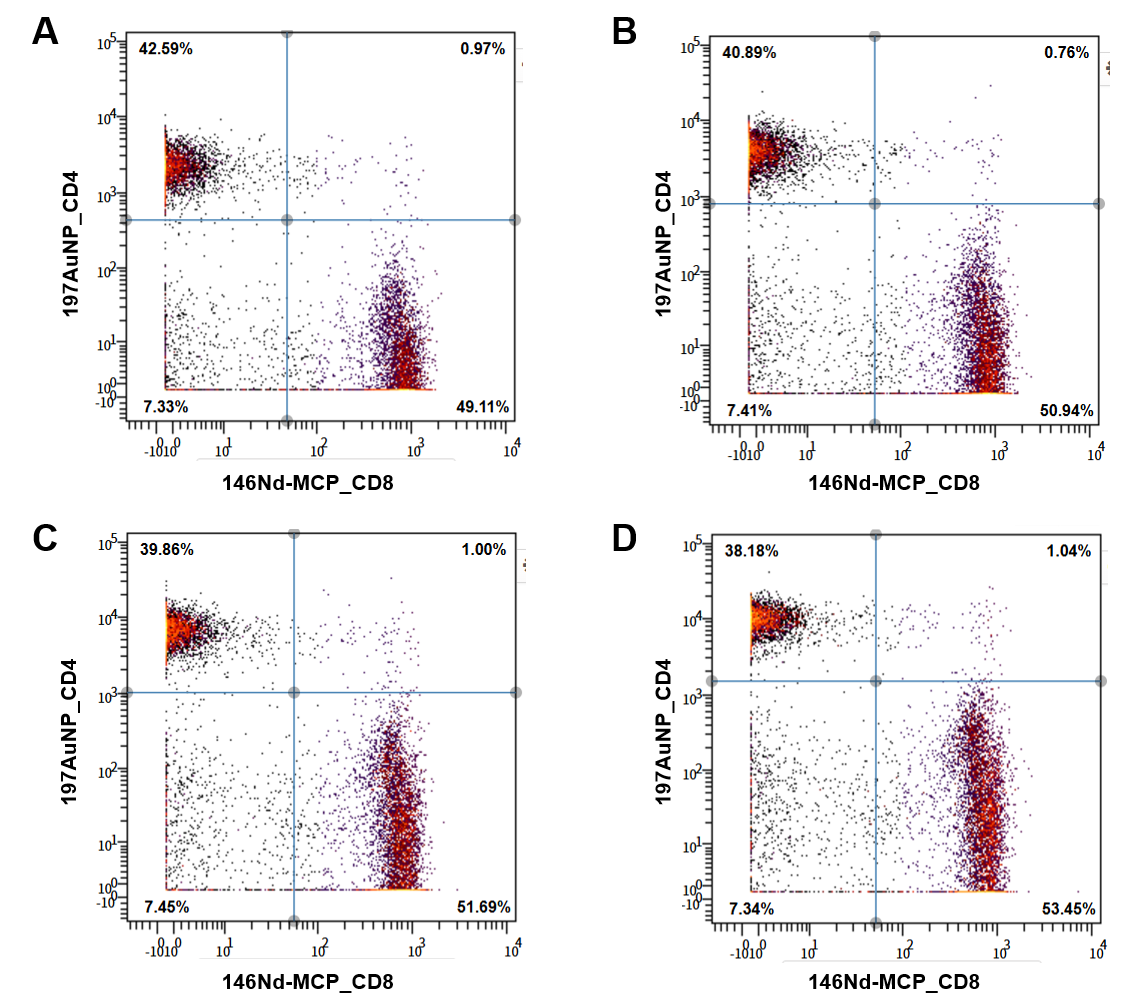


**Figure S5** The dot plots of CD4^+^/CD8^+^ T cell populations discriminated by 146Nd-MCP_CD8 and different dose of 197AuNP_CD4 (A-D, 17K-136K NPs/cell).


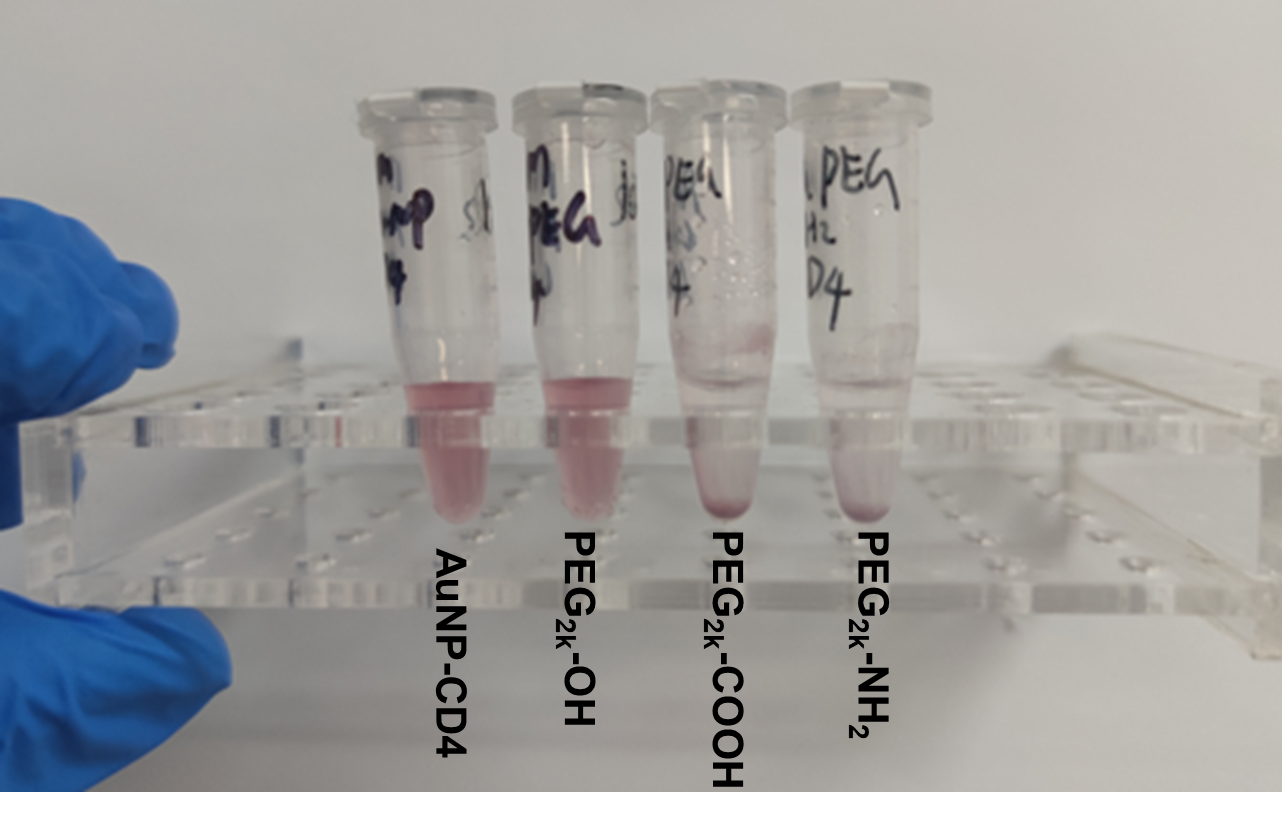


**Figure S6** AuNP_CD4 modified with different PEGs.


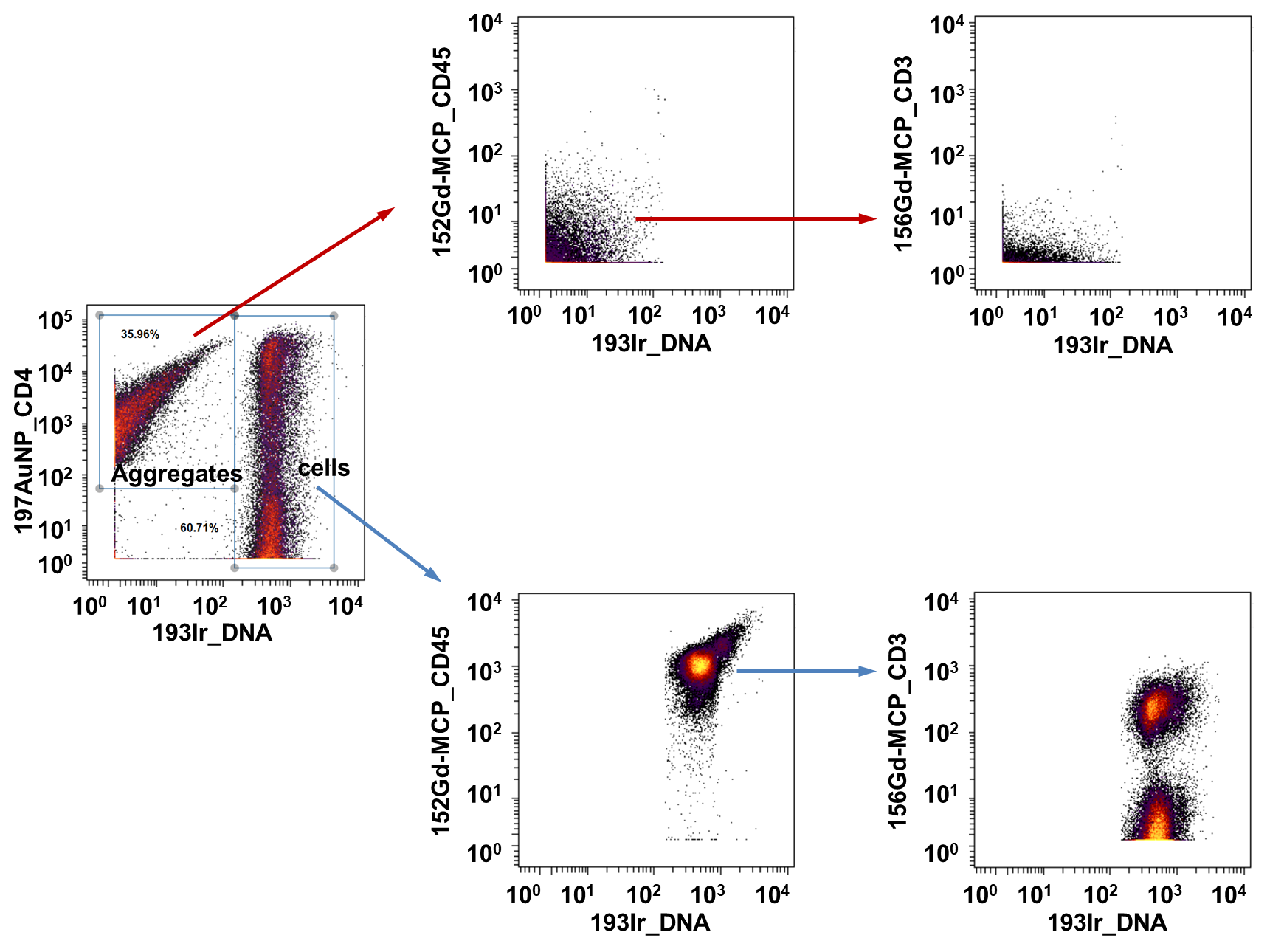


**Figure S7** The CD 45 and CD3 expression on aggregates and cells gates.


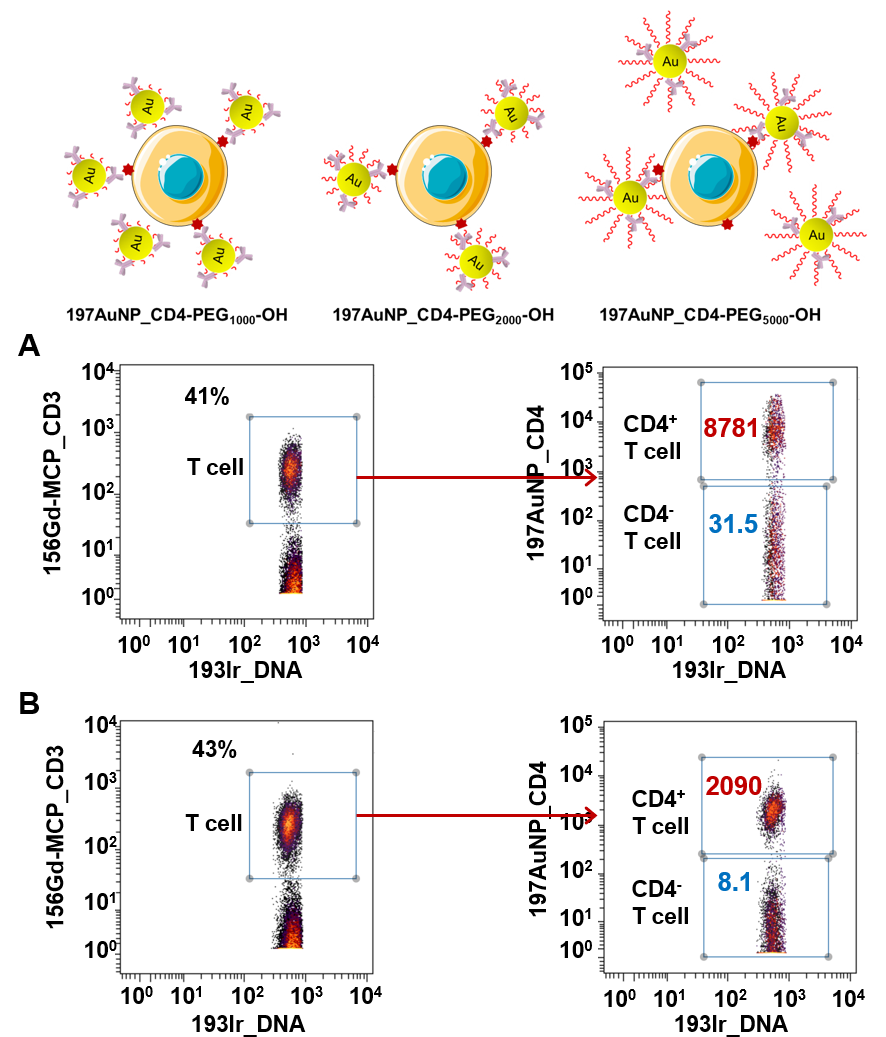


**Figure S8** The dot plots of T cells stained by (A)197AuNP_CD4-PEG_1000_-OH and (B)197AuNP_CD4-PEG_5000_-OH.


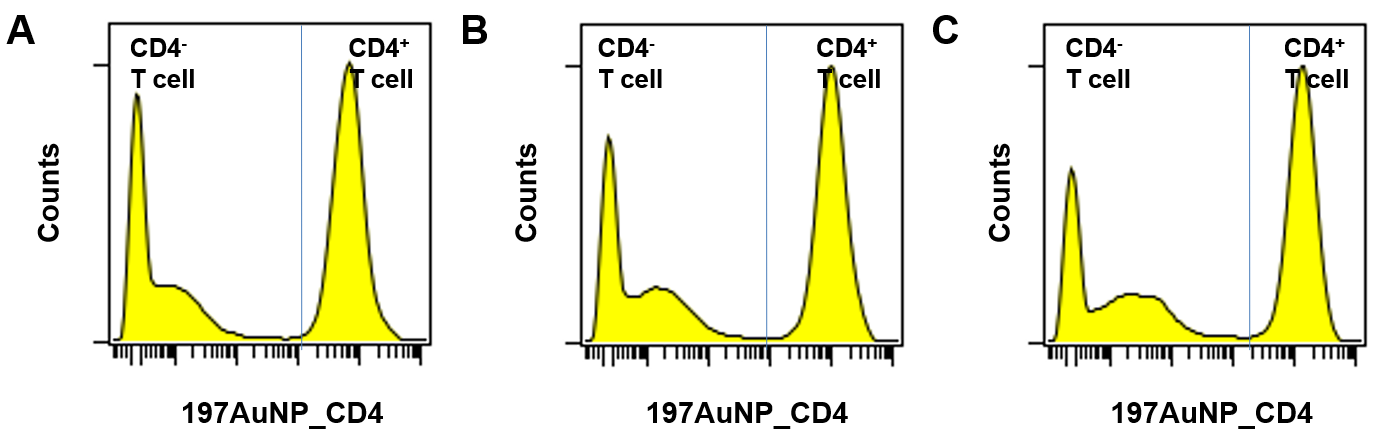


**Figure S9** The histograms of T cells stained by different dose of 197AuNP_CD4-PEG_2000_-OH (A) 136K NPs/cell; (B) 272K NPs/cell; (C) 544K NPs/cell.


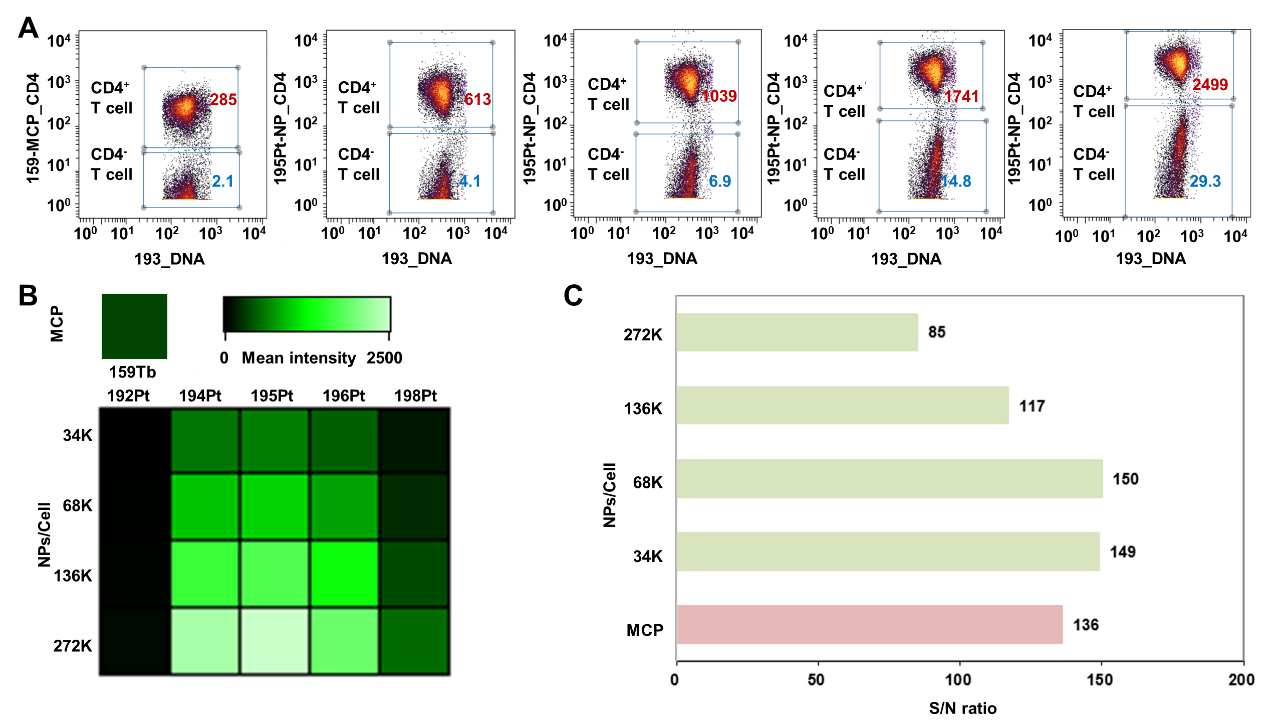


**Figure S10** (A) Dot plots and (B) heatmap of T cells stained by different dose of PtNP_CD4 (34K-272K NPs/cell); (C) The corresponding SB/NSB ratio.


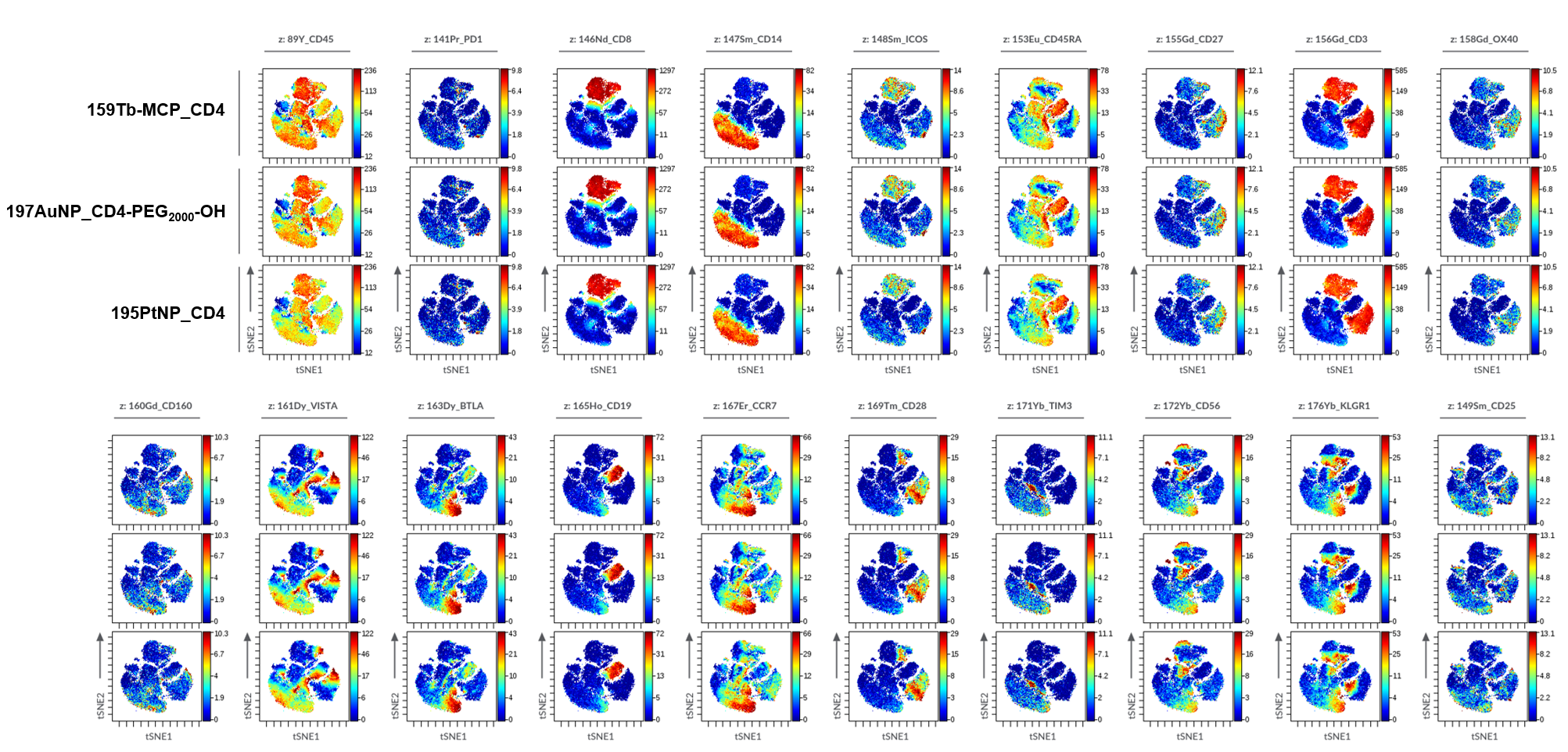


**Figure S11** The t-distributed stochastic neighbor embedding (t-SNE) dot plots depict protein expression of 19 surface markers of PBMCs stained with 159Tb-MCP_CD4, 197AuNP_CD4-PEG_2000_-OH and 195PtNP_CD4. Color bars range from blue to red, with red indicating elevated expression levels.


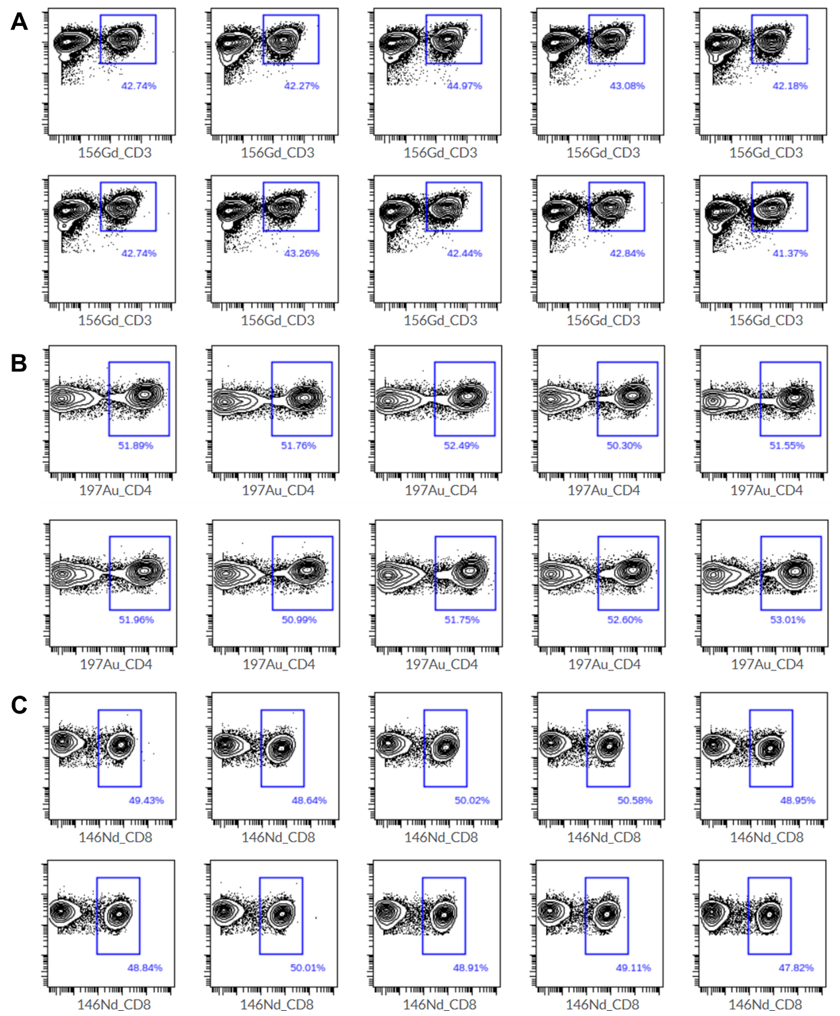


**Figure S12** Dot plots of (A) T cells; (B) CD4^+^ T cells and (C) CD8^+^ T cells in CV test.


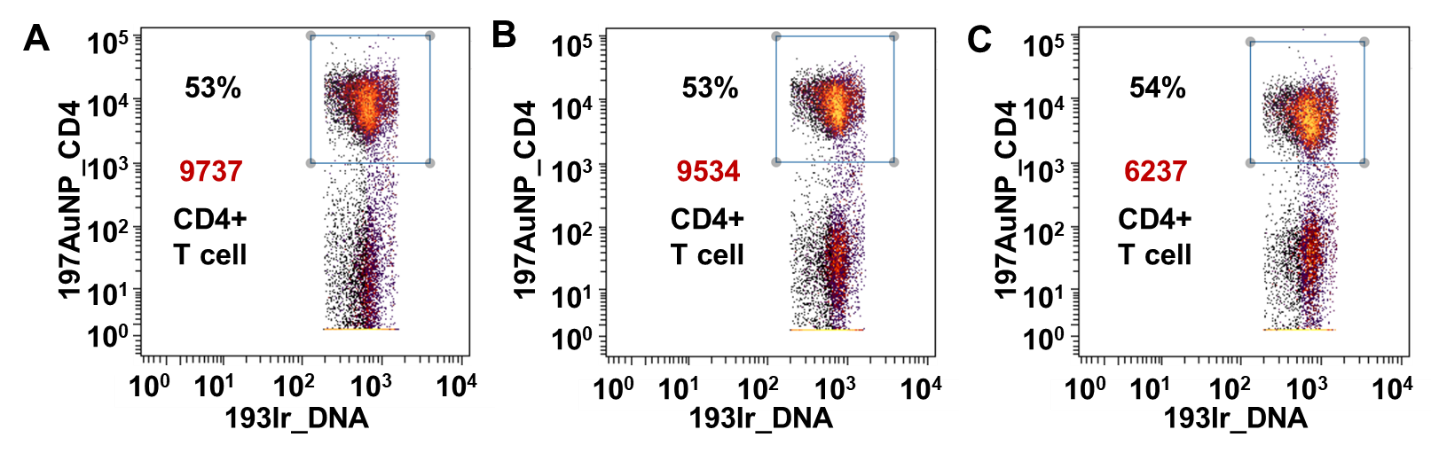


**Figure S13** The dot plots of T cells stained by197AuNP_CD4-PEG_2000_-OH (A) fresh, (B) after 30 day-storage and (C) after one year-storage.


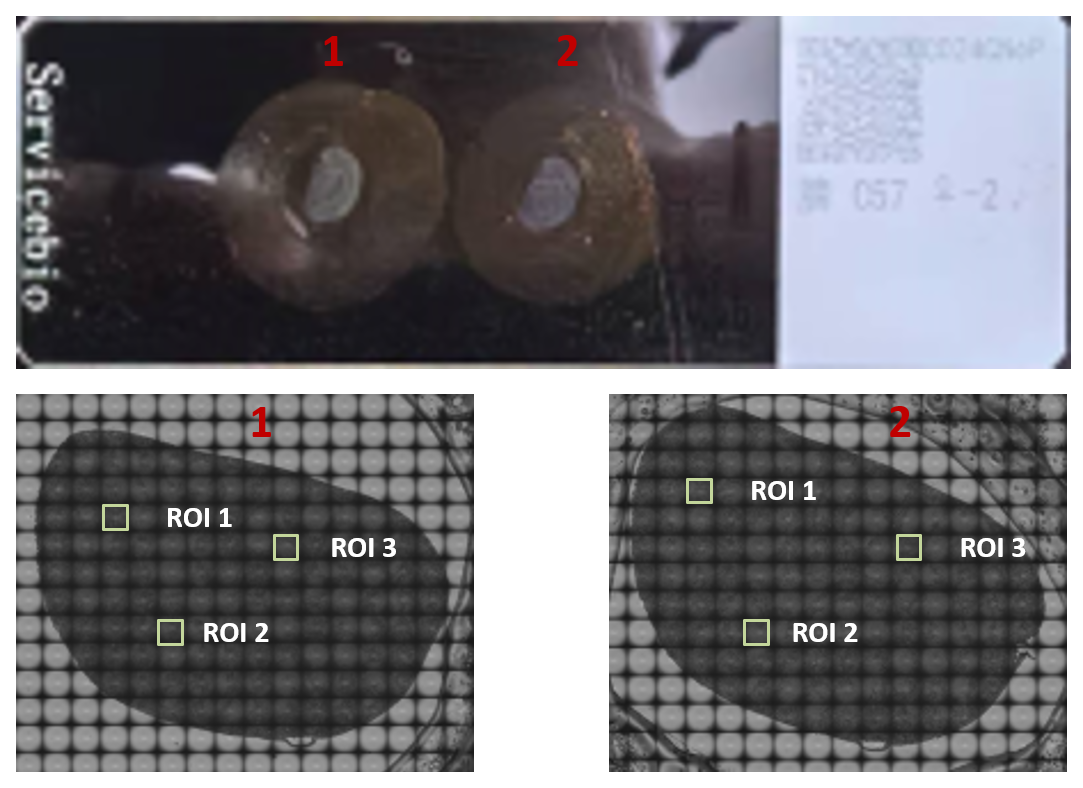


**Figure S14** Photographic image of two successive sections of a mouse spleen tissue. The two regions of interest (ROIs) are shown as yellow squares labeled ROI 1 and ROI 2 on the section on the right.


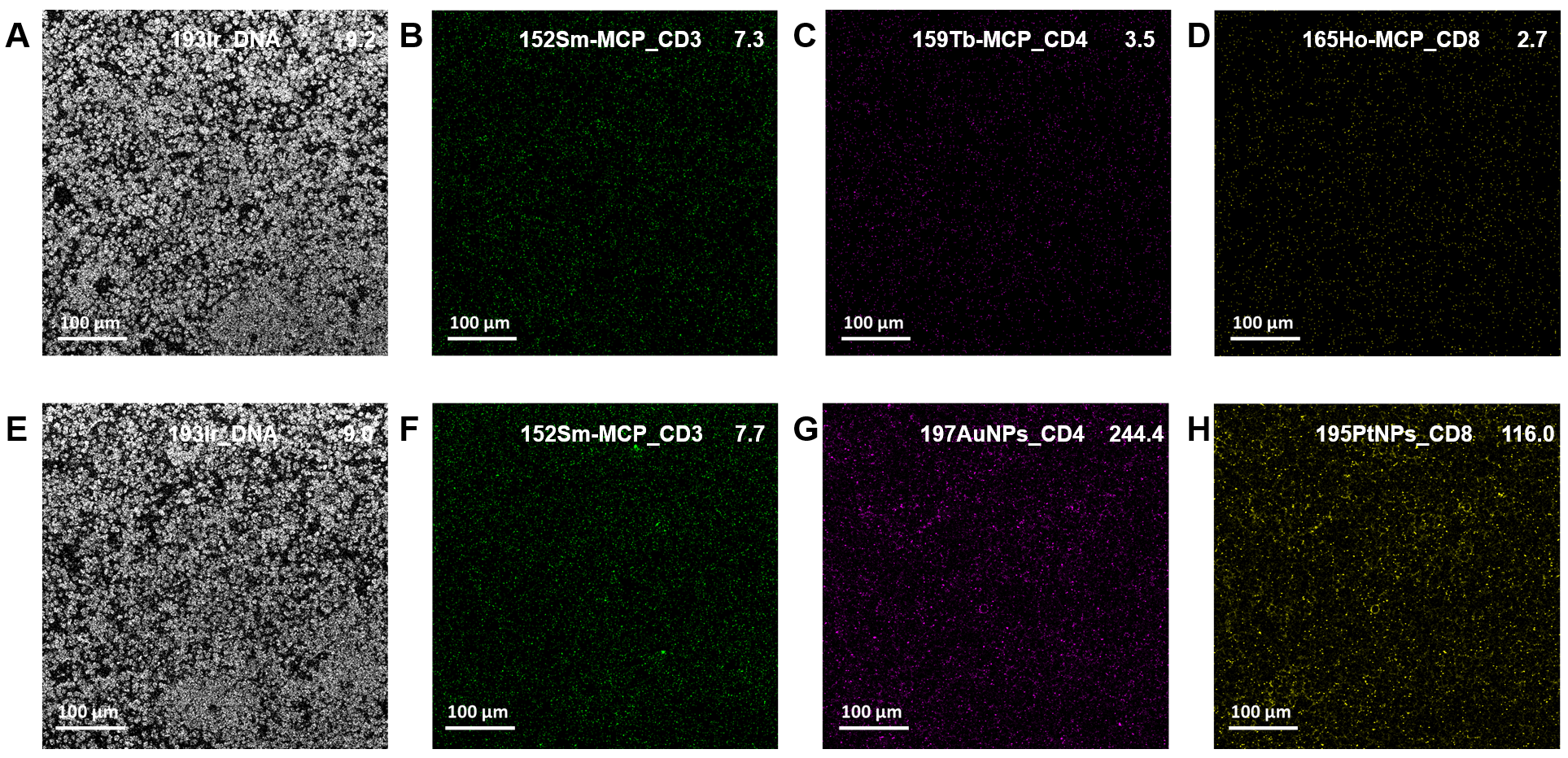


**Figure S1****5** IMC images for ROI 2. (A-D) white is assigned to 193Ir_DNA, lime is assigned to 152Sm-MCP_CD3, magenta is assigned to 159Tb-MCP_CD4 and yellow is assigned to 165Ho-MCP_CD8; (E-H) white is assigned to 193Ir_DNA, lime is assigned to 152Sm-MCP_CD3, magenta is assigned to 197AuNP_CD4-PEG_2000_-OH and yellow is assigned to 195PtNP_CD8. Scale bar shown on the lower left conner is 100 µm. The max threshold value is shown on the upper right corner.


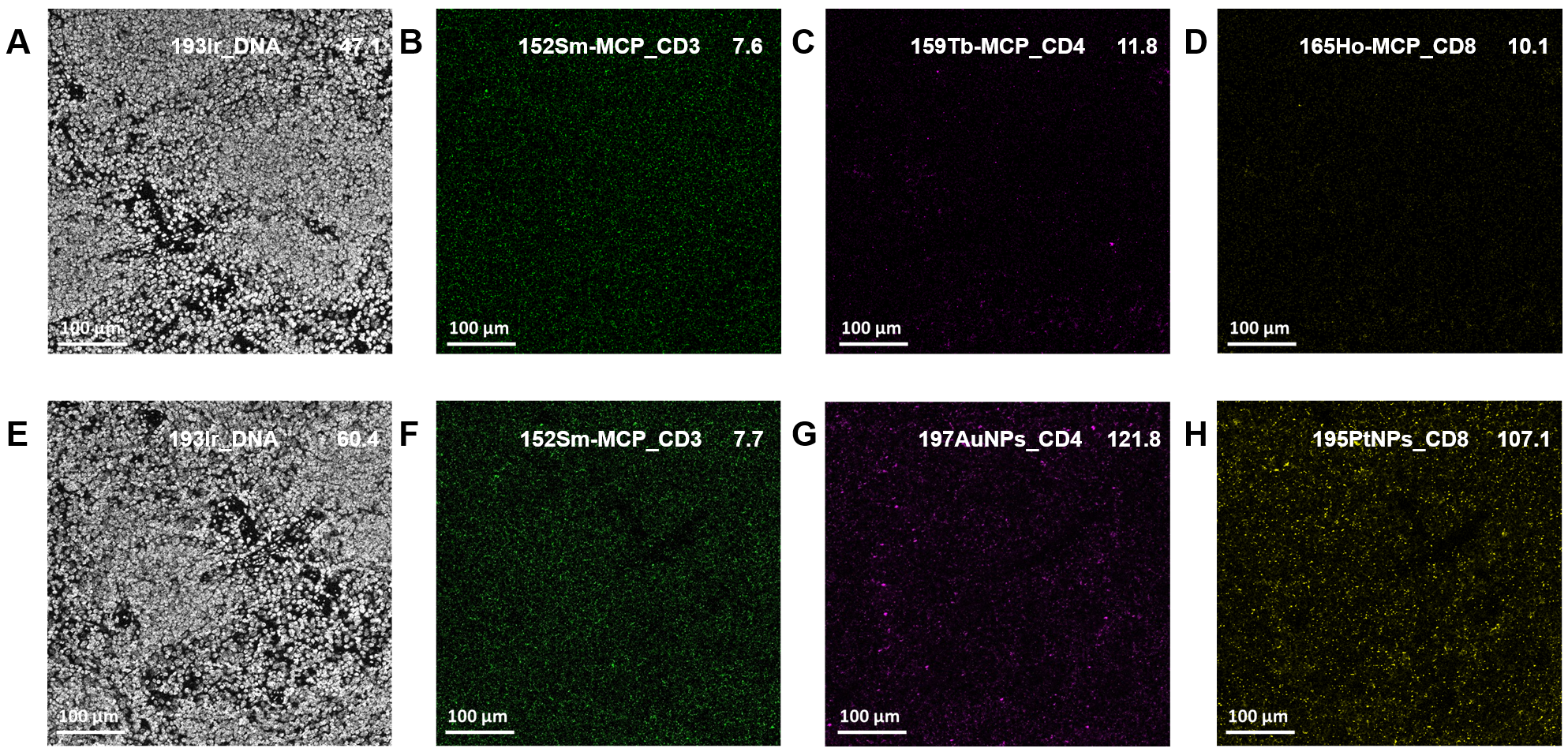


**Figure S16** IMC images for ROI 3. (A-D) white is assigned to 193Ir_DNA, lime is assigned to 152Sm-MCP_CD3, magenta is assigned to 159Tb-MCP_CD4 and yellow is assigned to 165Ho-MCP_CD8; (E-H) white is assigned to 193Ir_DNA, lime is assigned to 152Sm-MCP_CD3, magenta is assigned to 197AuNP_CD4-PEG_2000_-OH and yellow is assigned to 195PtNP_CD8. Scale bar shown on the lower left conner is 100 µm. The max threshold value is shown on the upper right corner.


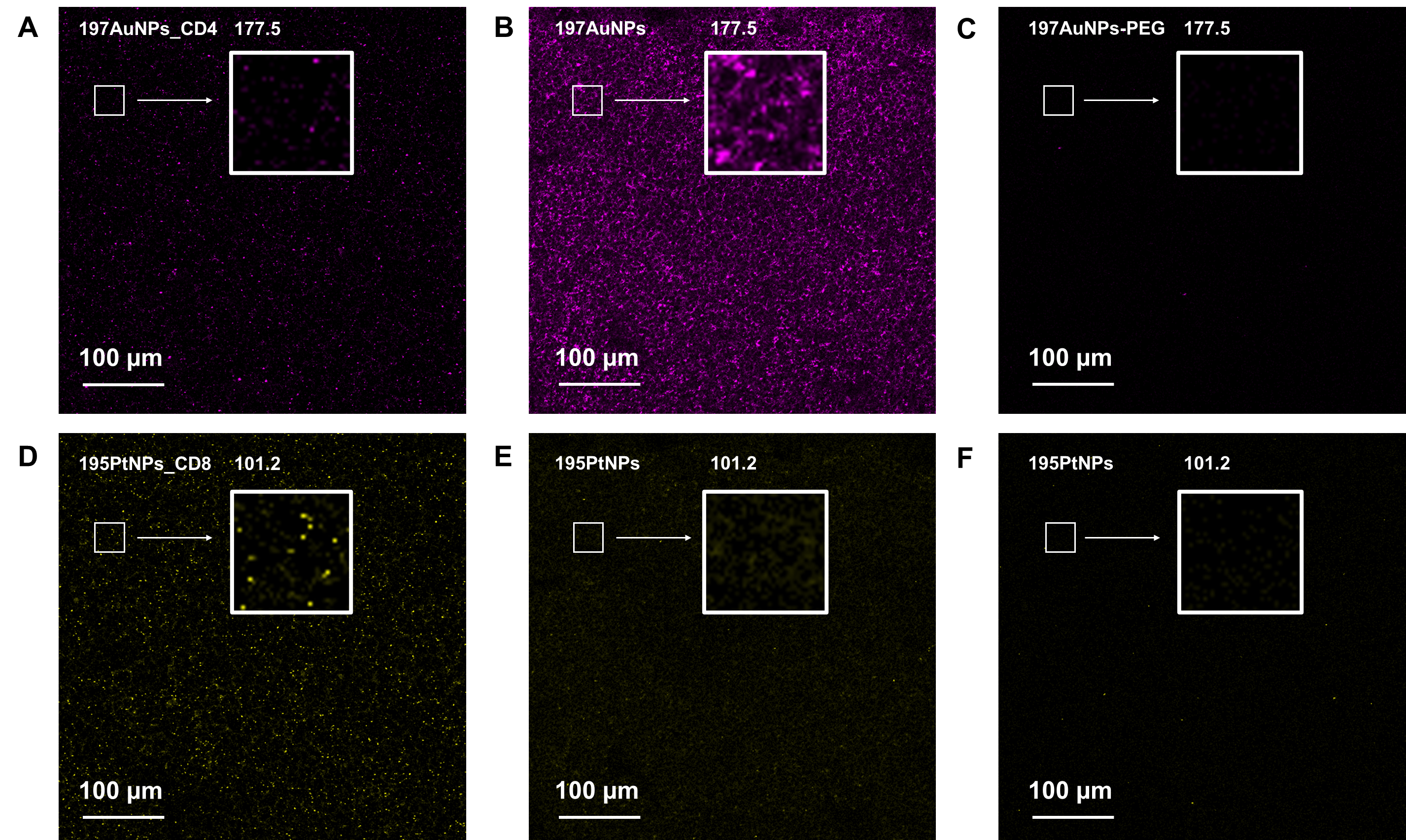


**Figure S17** IMC images for tissues stained by (A, D) 197AuNP_CD4-PEG_2000_-OH and 195PtNP_CD8; (B, E) 197AuNP and 195PtNP; (C, F) 197AuNP -PEG_2000_-OH and 195PtNP. Scale bar shown on the lower left conner is 100 µm. The max threshold value is shown on the upper middle.


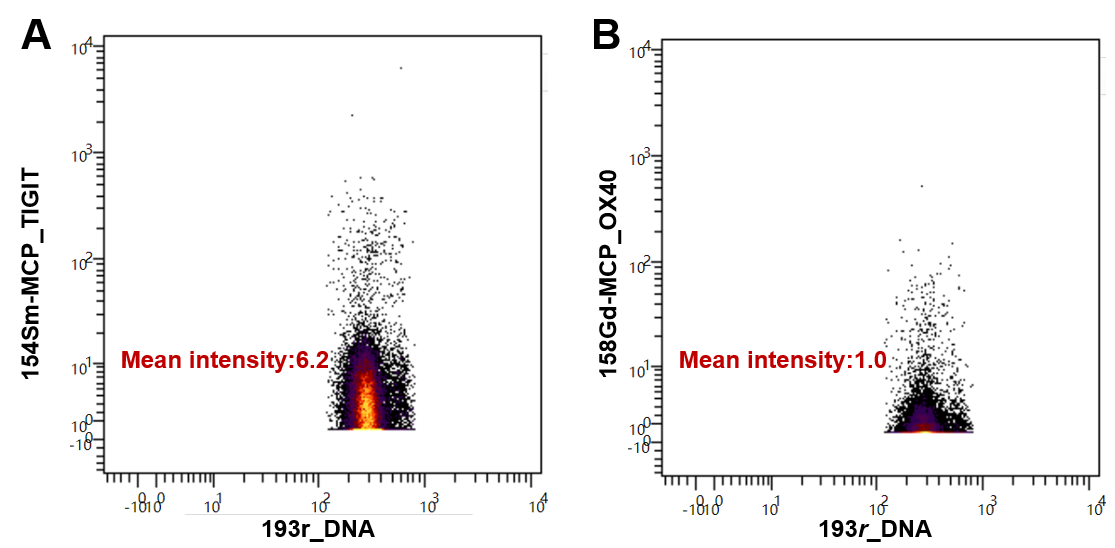


**Figure S18** The dot plots of PBMC stained by 154Sm-MCP_TIGIT (A) and 158Gd-MCP_OX40 (B).


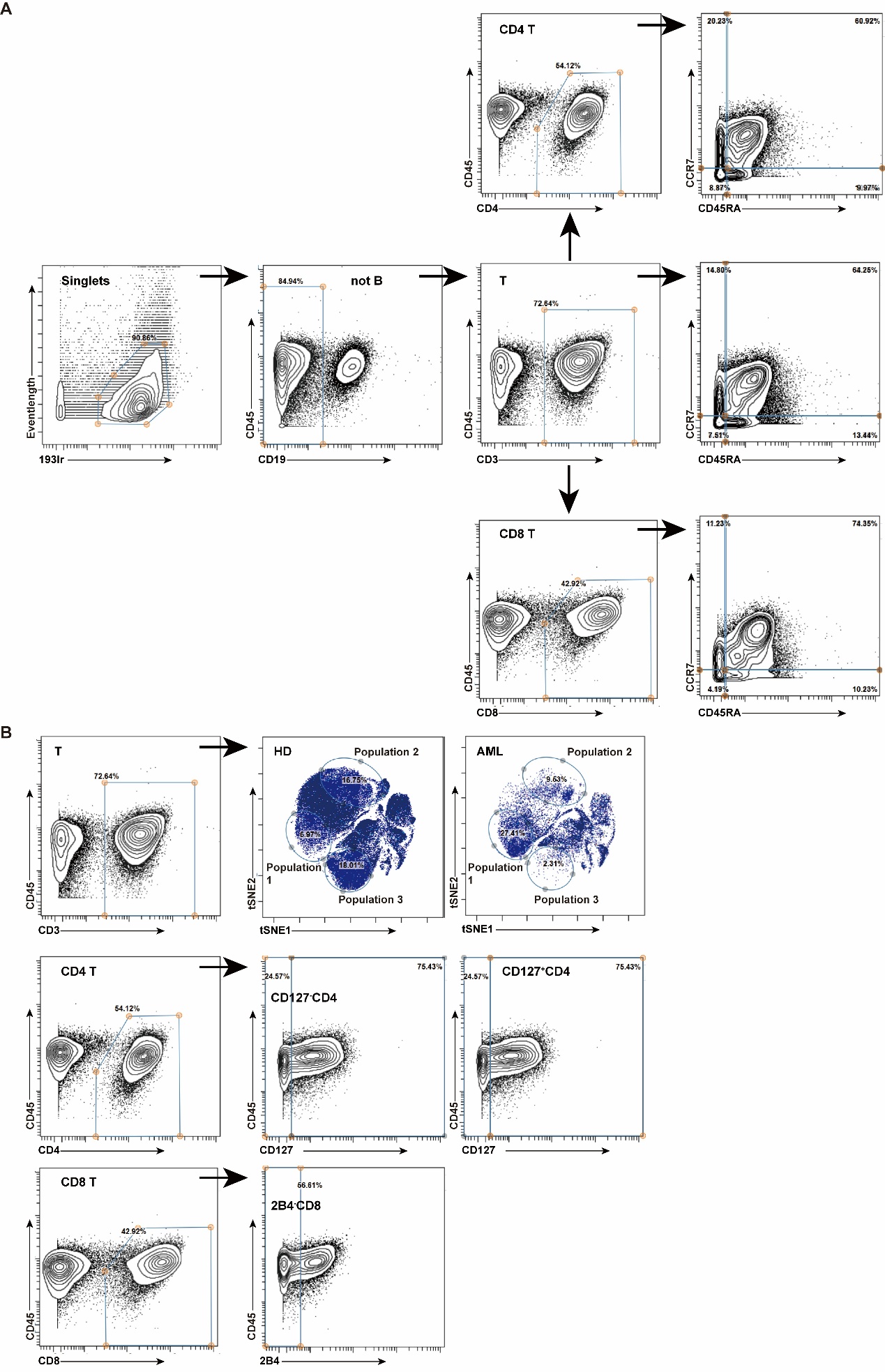


**Figure S19** Gating strategy for population 1-3.


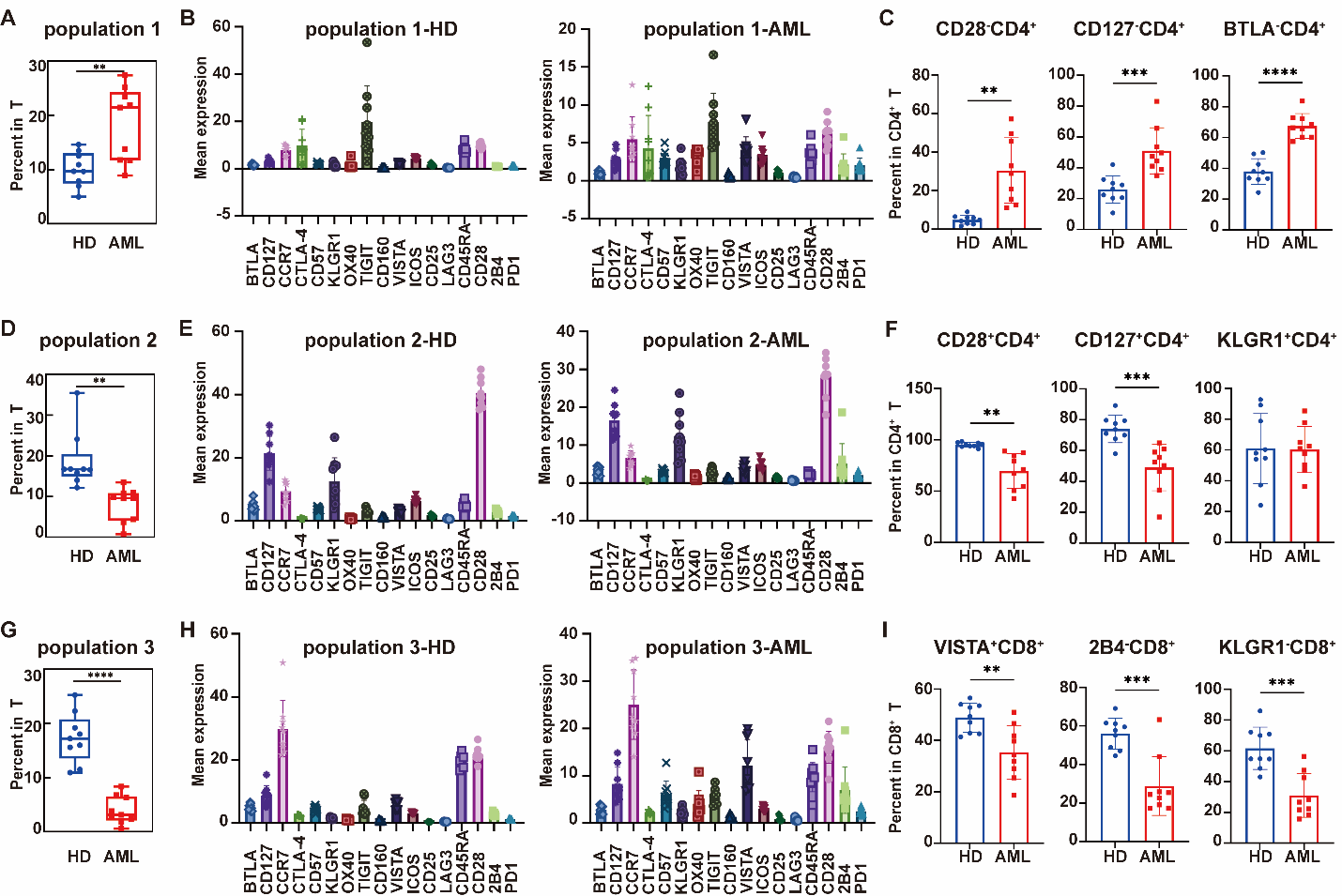


**Figure S20** (A, D, G) Bar plots showing the significantly changed frequencies of populations 1-3 in T cells; (B, E, H) Mean expression signatures of indicated populations in HD and AML; (C, F, I) Corresponding bar plots showing statistically significant changed frequencies of indicated cell subpopulations.

# Supporting Tables

**Table S1** The population distributions (%) and mean intensity of gated cell subgroups stained by 156Gd-MCP_CD3, AuNP_CD4-PEG_2000_-OH, and 146Nd-MCP_CD8.

|  | CD3^+^ cells  (%) | MSI | CD4^+^ T cells  (%) | MSI | CD8^+^ T cells  (%) | MSI |
| --- | --- | --- | --- | --- | --- | --- |
| 1 | 42.74 | 305 | 51.89 | 7293 | 49.43 | 787 |
| 2 | 42.27 | 247 | 51.76 | 5794 | 48.64 | 623 |
| 3 | 44.97 | 271 | 52.49 | 6085 | 50.02 | 714 |
| 4 | 43.08 | 286 | 50.30 | 6840 | 50.58 | 777 |
| 5 | 42.18 | 240 | 51.55 | 6731 | 48.95 | 638 |
| 6 | 42.74 | 268 | 51.96 | 6844 | 48.84 | 720 |
| 7 | 43.26 | 277 | 50.99 | 6870 | 50.01 | 752 |
| 8 | 42.44 | 254 | 51.75 | 6615 | 48.91 | 692 |
| 9 | 42.84 | 277 | 52.60 | 6842 | 49.11 | 707 |
| 10 | 41.37 | 243 | 53.01 | 6622 | 47.82 | 669 |
| CV | 2.18% | 7.81% | 1.52% | 6.40% | 1.63% | 7.75% |

**Table S2** Comparison of literature reports for nanoparticle typed mass tags

| Mass tag | Metal atom capacity | Signal amplification (fold) | Minimum dose for cell clustering (NPs/cell) | SB/NSB ratio |
| --- | --- | --- | --- | --- |
| AuNP_CD4-PEG_2k_-OH (This work) | 31000 | 48 | 34 | 938 |
| PEGylated NaHoF_4__CD14^a^ | 12000 | 30 | 130 | 570 |
| MPF_PD-1^b^ | 1300 | 9 | 15000 | 67 |
| Zr-MOF_CD45^c^ | 1×10^5^ | 5 | 1500 | 75 |
| TaO_2__CD25^d^ | 2700 | 9 | NA | 200 |
| AgNP_CD25^e^ | 2×10^6^ | 3 | NA | NA |

NA: not mentioned in the publications.

**Table S3** Demographic characteristics of mass cytometry datasets.

| Groups  N  Age (years)  Sex (F/M) | HD  13  27.8±3.4  3/10 | AML  19  53.5±12.4  10/9 |
| --- | --- | --- |
| Note:  HD: healthy donor;  AML: AML patients;  Data are expressed as mean ± SD. | | |

**Table S4** Antibodies used in panel for HD and AML examination with the metal conjugate, clone and manufacturer

| **Antigen** | **Conjugate** | **Clone** | **Manufacturer** |
| --- | --- | --- | --- |
| CD45 | 89-Y | HI30 | Biolegend |
| PD-1 | 141-Pr | EH12.2H7 | Biolegend |
| CD14 | 147-Sm | HCD14 | Biolegend |
| ICOS | 148-Nd | C398.4A | Biolegend |
| CD25 | 149-Sm | S20019C | Biolegend |
| LAG3 | 150-Nd | 11C3C65 | Biolegend |
| CD3 | 152-Sm | UCHT1 | Biolegend |
| CD45RA | 153-Eu | HI100 | Biolegend |
| 2B4 | 156-Gd | C1.7 | Biolegend |
| CD19 | 158-Gd | HIB19 | Biolegend |
| CD4 | 159-Tb | SK3 | Biolegend |
| CD160 | 160-Gd | BY55 | Biolegend |
| VISTA | 161-Dy | A17002B | Biolegend |
| BLTA | 163-Dy | 80H3 | Biolegend |
| CD127 | 165-Ho | A019D5 | Biolegend |
| CD197/CCR7 | 167-Er | 3D12 | BD |
| CD8 | 168-Er | HIT8a | Biolegend |
| CD28 | 169-Tm | CD28.2 | BD |
| CTLA-4 | 170-Er | L3D10 | Biolegend |
| CD56 | 172-Yb | 5.1H11 | Biolegend |
| CD57 | 175-Lu | HNK-1 | Biolegend |
| KLGR1 | 176-Yb | SA231A2 | Biolegend |
| OX40 | 195-Pt | Ber-ACT35 | Biolegend |
| TIGIT | 197-Au | A15153G | Biolegend |

# References

a Pichaandi, J.; Zhao, G.; Bouzekri, A.; Lu, E.; Ornatsky, O.; Baranov, V.; Nitz, M.; Winnik, M. A., Lanthanide nanoparticles for high sensitivity multiparameter single cell analysis. *Chem. Sci.* **2019,** *10* (10), 2965-2974.

b Chen, Y.; Wang, G.; Wang, P.; Liu, J.; Shi, H.; Zhao, J.; Zeng, X.; Luo, Y., Metal-Chelatable Porphyrinic Frameworks for Single-Cell Multiplexing with Mass Cytometry. *Angew. Chem. Int. Edit.* **2022,** *61* (38), e202208640.

c Dang, J.; Li, H.; Zhang, L.; Li, S.; Zhang, T.; Huang, S.; Li, Y.; Huang, C.; Ke, Y.; Shen, G.; Zhi, X.; Ding, X., New Structure Mass Tag based on Zr-NMOF for Multiparameter and Sensitive Single-Cell Interrogating in Mass Cytometry. Adv. Mater. 2021, 33 (35), 2008297.

d Zhang, Y.; Zabinyakov, N.; Majonis, D.; Bouzekri, A.; Ornatsky, O.; Baranov, V.; Winnik, M. A., Tantalum Oxide Nanoparticle-Based Mass Tag for Mass Cytometry. Anal. Chem. 2020, 92 (8), 5741-5749.

e Schulz, A. R.; Stanislawiak, S.; Baumgart, S.; Grützkau, A.; Mei, H. E., Silver nanoparticles for the detection of cell surface antigens in mass cytometry. Cytom. Part. A 2017, 91 (1), 25-33.
